# Mechanistic modelling of tyrosine recombination reveals key parameters determining the performance of a CAR T cell switching circuit

**DOI:** 10.1049/enb.2019.0020

**Published:** 2020-03-24

**Authors:** Jack E. Bowyer, Deboki Chakravarti, Wilson W. Wong, Declan G. Bates

**Affiliations:** ^1^ School of Engineering University of Warwick Coventry CV4 7AL UK; ^2^ Warwick Integrative Synthetic Biology Centre Coventry CV4 7AL UK; ^3^ Department of Biomedical Engineering Boston University Boston MA 02215 USA; ^4^ Biological Design Center Boston University Boston MA 02215 USA

**Keywords:** molecular biophysics, gene therapy, biochemistry, drugs, genetic engineering, biomedical materials, tumours, DNA, enzymes, cancer, cellular biophysics, statistical approximation approaches, system parameters, optimal switch performance, switching response, inducer drug 4‐OHT, FlpO recombinase, mechanistic modelling, tyrosine recombination, CAR T cell, inducible genetic switches, tyrosine recombinase‐based DNA excision, chimeric antigen receptor T cell activity, cancer immunotherapy, increased stability, tyrosine recombinases, inversion–excision circuit design, mechanistic mathematical model

## Abstract

Inducible genetic switches based on tyrosine recombinase‐based DNA excision are a promising platform for the regulation and control of chimeric antigen receptor (CAR) T cell activity in cancer immunotherapy. These switches exploit the increased stability of DNA excision in tyrosine recombinases through an inversion–excision circuit design. Here, the authors develop the first mechanistic mathematical model of switching dynamics in tyrosine recombinases and validate it against experimental data through both global optimisation and statistical approximation approaches. Analysis of this model provides guidelines regarding which system parameters are best suited to experimental tuning in order to establish optimal switch performance in vivo. In particular, they find that the switching response can be made significantly faster by increasing the concentration of the inducer drug 4‐OHT and/or by using promoters generating higher expression levels of the FlpO recombinase.

## 1 Introduction

Cell‐based therapies that employ engineered T cells expressing chimeric antigen receptors (CARs) to target cancer cells have demonstrated promising responses in recent clinical trials, especially with regard to B cell cancers [1, 2, 3, 4]. T cell immunotherapy involves extracting T cells from a patient's blood, engineering the cells to improve their ability to detect and target tumour cells, and reintroducing them back into the patient. Synthetic CARs are integrated into the cells which enable them to recognise specific tumour‐associated antigens. There are, however, a number of significant safety and efficacy concerns that have arisen regarding T cell therapies. Success against solid tumours has been limited due to difficulties relating to autoreactive ‘on‐target, off‐tumour’ responses as well as cytokine release syndrome, a response to antigen stimulation that accelerates the immune response to potentially fatal levels. Strategies such as kill switches, designed to terminate the T cell response in cases of high toxicity, terminate the entire therapy which often leads to the continuation of tumour development. Reliable modulation of the therapy is therefore of great importance and has prompted research in the development of switching and control circuitry to enable the regulation of engineered T cell responses [5, 6, 7, 8, 9, 10, 11, 12, 13, 14, 15, 16, 17].

A promising approach for the creation of such circuitry is the use of site‐specific recombinases (SSRs), which are capable of precise DNA manipulation in mammalian cells [18, 19, 20], and can be used to engineer synthetic biological circuits capable of performing user‐defined functions that are programmed into the cellular DNA [21, 22]. Moreover, SSR‐based gene switches elicit permanent alterations to the DNA and thus possess a built‐in memory property. As such, SSR‐based gene switches are capable of stable changes in gene expression after transient exposure to drug inducer. This memory property could simplify the implementation of such circuitry in gene therapy or cellular immunotherapy, where continuous drug dosing to control transgene expression in patients presents a logistic challenge.

SSRs belong to two main families, the serine and tyrosine recombinases. The former mediate unidirectional (irreversible) recombination events that produce stable genetic states [23, 24], however, the reversal of recombination events via serine integrases is typically dependent on a recombination directionality factor (RDF) [25, 26, 27, 28], which can compromise their efficiency [29, 30]. Consequently, serine recombinase‐based circuit designs attempt to exploit unidirectional recombination events that are, therefore, unable to facilitate transitioning back to prior system states [31]. Recent mathematical modelling studies regarding serine integrases and their RDFs have revealed that computational models are capable of quantitative replication of the observed in vitro dynamics [30, 32] and can aid the examination of the practical viability of novel synthetic circuits that combine multiple biological elements [30, 33]. Here we seek to develop the first equivalent model for tyrosine recombinase‐based circuitry and explore its use in determining key experimental parameters that govern the performance of a CAR T cell switching circuit.

**Fig. 1:**
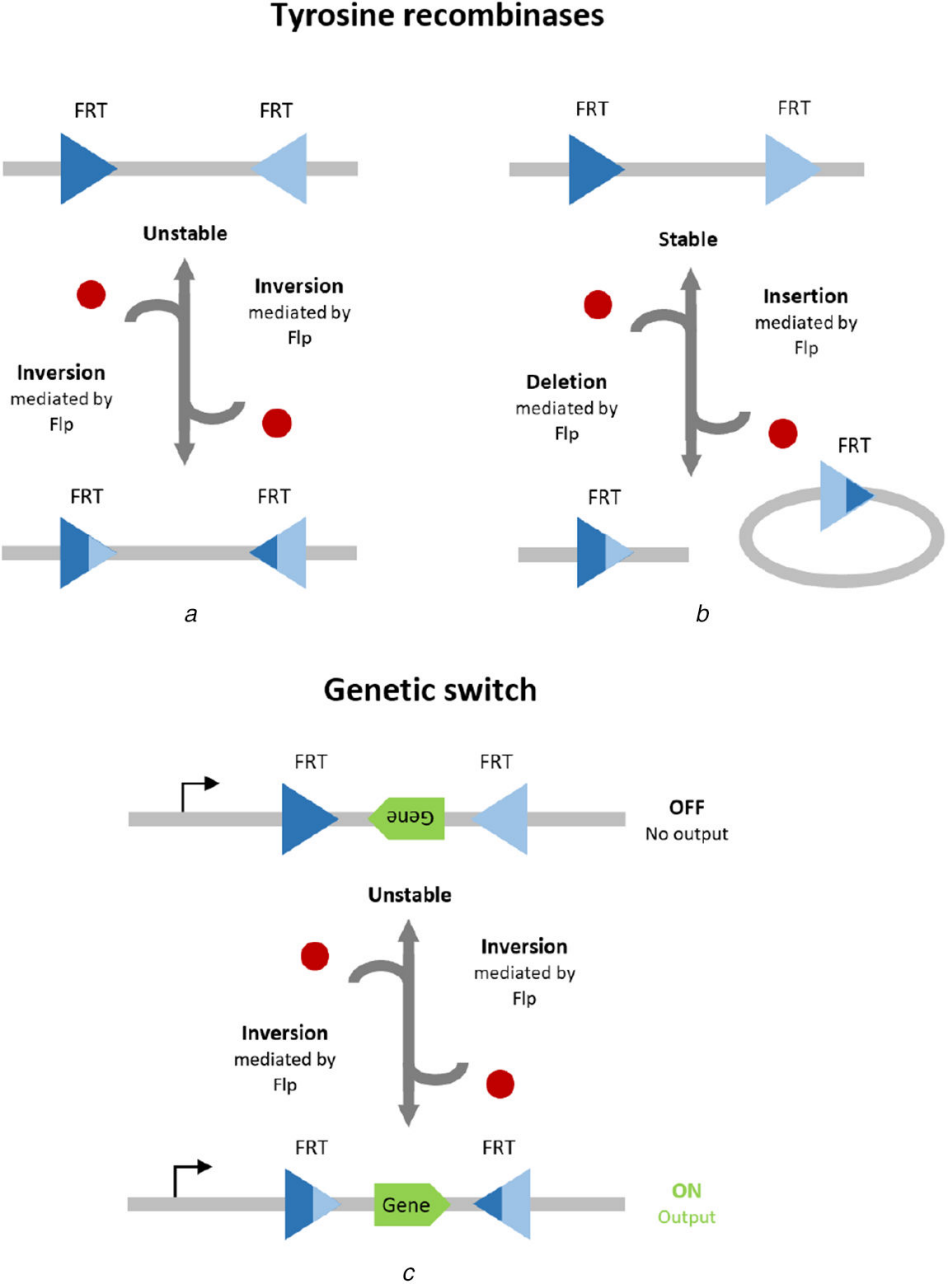
Schematic diagrams of DNA recombination events mediated by tyrosine recombinases ***(a)*** Antiparallel FRT sites result in reversible DNA inversion results, ***(b)*** Parallel FRT sites result in reversible DNA excision with increased stability, ***(c)*** Elementary inversion‐based tyrosine recombinase circuit design is not viable for reliable control over gene expression

Tyrosine recombinases elicit a primary inversion event in an identical manner to that of serine integrases. The secondary inversion event, however, can also be mediated by the same tyrosine recombinase without any additional cofactors or RDFs, which causes bidirectionality (reversibility) (Fig. 1*a*) [34, 35]. Tyrosine recombinase binding interactions exhibit positive cooperativity [19, 36, 37] and comprise a series of sequential single strand exchanges known as a Holliday junction [38, 39]. Deletion and insertion events mediated by tyrosine recombinases also mirror that of their serine cousins, with the exception of bidirectionality (Fig. 1*b*). Hence, adopting an elementary inversion switch design is not viable, since inversion events are susceptible to natural reversibility, which compromises the efficiency of the desired transcriptional response (Fig. 1*c*). Reversibility is theoretically more prominent in inversion events compared to deletion and insertion events. This is because the deletion of a genetic sequence forms a loop of DNA that dissociates from its original strand, and therefore presents additional spatial disparity to overcome in a subsequent insertion event, compared to successive inversions of the same genetic sequence. Consequently, DNA excision has emerged as a viable method for developing tyrosine recombinase‐based circuitry [40, 41]. The inducible control over gene expression afforded by SSRs has the potential to significantly improve the efficacy of engineered T cells. Recombinase‐based genetic switches represent the most versatile switches in T cells, which have the potential to enable optimal T cell activity that can be tuned via drug dosage and duration, whilst retaining the memory of transient stimuli.

Mathematical models have been used to elucidate a wide variety of synthetic biological circuits. These include gene regulatory networks [42, 43], tuneable oscillators [44], and genetic counters [21]. Problems that arise as a side effect of assembling synthetic biological circuits can also be addressed using mathematical models such as the burden placed on cell growth due to the introduction of synthetic constructs [45]. At the very least, *in silico* simulation of a given circuit can test hypotheses relating to its dynamic performance in a fraction of the time taken to carry out the equivalent experimental study. The development of new models, therefore, has the potential to create a host of valuable tools for synthetic biologists looking to design and implement novel circuitry in vivo [46]. In this study, we present a mechanistic mathematical model of an inversion–excision switch based on our knowledge of the underlying molecular interactions associated with tyrosine recombinases. The model is formulated through the application of mass action kinetics, thus providing a deterministic output via numerical simulation. The model is validated through two separate data‐fitting procedures. The first is a global optimisation technique that minimises error through a ‘survival of the fittest’ procedure. The second is a statistical technique that employs Bayes’ theorem to infer the parameterisation most likely to have produced the data. Our validated model is used to demonstrate an inverse proportionality between 4‐OHT concentration and switch response time and identify which model parameters are most sensitive to perturbations and are hence best suited to experimental tuning in vivo.

## 2 Results and discussion

### 2.1 Mechanistic model of a tyrosine recombinase inversion–excision switch

**Fig. 2:**
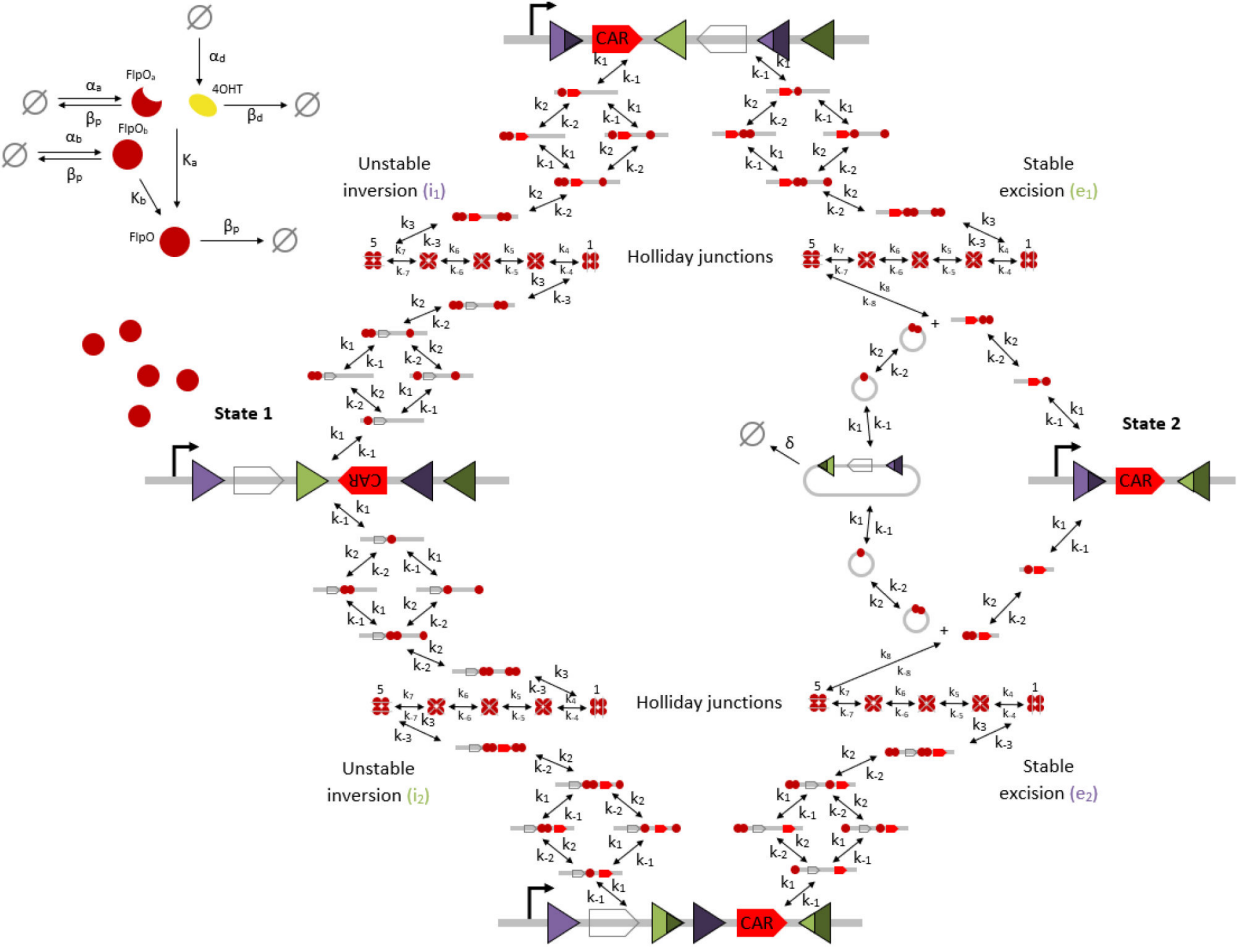
Schematic diagram of a tyrosine recombinase‐based inversion–excision switch. FlpO recombinase in its activated form targets orthogonal pairs of FRT attachment sites in transitioning the switch from State 1 to State 2 via two transient intermediate genetic states. FRT sites are depicted as purple and green triangles with different shades used to illustrate the result of recombination events. Clear and red pointed boxes depict the blank sequence and CAR gene, respectively. Black arrows depict DNA:protein binding reactions comprising unstable inversion and stable excision events. The rate of unactivated FlpO expression is denoted by α_a_; the rate of expression of activated FlpO that leaks into the nucleus is denoted by α_b_; the concentration of 4‐OHT in the system is modelled as an expression rate denoted by α_d_ and is an initial condition of the model. The rate of degradation of the recombinase protein is denoted by β_p_; the rate of degradation of drug inducer is denoted by β_d_. The rate of FlpO activation and nuclear localisation is denoted by K_a_; the rate of leaky FlpO nuclear localisation is denoted by K_b_. The rate of a FlpO monomer binding reversibly to free FRT sites is denoted by k_1_, k_−1_; due to the cooperativity of FlpO monomer binding, we denote the rate of a FlpO monomer binding reversibly to an occupied FRT site by k_2_, k_−2_. The parameters k_1_, k_−1_, k_2_, k_−2_ are equal for each pair of recombination events involving the same pair of attachment sites, i.e. the i_1_ and e_2_ parameters are equal and the i_2_ and e_1_ parameters are equal. The rate of Holliday junction formation is denoted by k_3_, k_−3_ for the inversions reactions, and by k_3_, k_−3_, k_8_, k_−8_ for the excision reactions. Each of the five Holliday junction strand exchanges is denoted by k_4_, k_−4_, k_5_, k_−5_, k_6_, k_−6_, k_7_, k_−7_, respectively. The rate of dilution of the excised DNA (S_X_) due to cell division is denoted by δ. The empty set symbol is used to depict expression and degradation reactions

The tyrosine recombinase genetic switch developed in [41] exploits the increased stability of DNA excision through a gated inversion–excision circuit design (Fig. 2). The input to this circuit is the flippase O (FlpO) recombinase, an optimised variant of flippase (Flp) with increased efficiency within mammalian cells [47]. A metabolite of the Food and Drug Administration‐approved drug tamoxifen, known as 4‐OHT, is used to activate the FlpO before it can enter the nucleus and mediate the necessary DNA recombination events. FlpO is expressed constitutively in the cytosol until the advent of 4‐OHT causes activation and transport into the nucleus. Leaky nuclear localisation of FlpO can also occur in the absence of 4‐OHT, resulting in basal switch activity.

The full network of molecular interactions underlying the switch is shown in Fig. 2, and consists of each DNA:protein and Holliday junction complex arising from FlpO binding interactions. The initial system state (State 1) consists of a blank sequence flanked by antiparallel FRT sites and an inverted CAR gene flanked by parallel FRT sites. This setup does not provide any CAR expression since the non‐expressing blank sequence is positioned immediately downstream of the promoter and the CAR gene is not suitably oriented for transcription. FlpO expression elicits inversion of either FRT site pair to produce two transient genetic states. The first sees inversion of both genes, resulting in the CAR expression due to the now suitably oriented CAR gene immediately downstream of the promoter. The second sees the inversion of the CAR gene downstream of the blank sequence which also results in CAR expression. Both transient states consist of the blank sequence flanked by parallel FRT sites, and subsequent FlpO binding, therefore, elicits excision events that delete this sequence. Excision results in a shortened DNA sequence that consists of the CAR gene downstream of the promoter, which therefore provides CAR expression (State 2). In summary, State 1 provides no gene expression, the transient states both provide CAR expression and State 2 also provides CAR expression.

There are four molecular reaction networks comprising the inversion–excision switch system; two unstable inversion networks that detail the transition from State 1 to the two transient genetic states (*i*_1_, *i*_2_), and two stable excision networks that detail the transition from the transient genetic states to State 2 (*e*_1_, *e*_2_). The inversion reaction network is symmetrical due to the fact that successive inversion events are identical. Free DNA is bound by monomeric FlpO until two monomers associate with each of the corresponding attachment sites whereby inversion is initiated. We account for the cooperativity of monomer binding by modelling two distinct DNA:protein complexes with one FlpO monomer bound at each attachment site ($D_{S_{p}}^{i_{n}/e_{m}}F_{1,1}$) and two monomers bound at one site ($D_{S_{p}}^{i_{n}/e_{m}}F_{2,0}$). Details of the mathematical representation of cooperativity in our model can be found in Section 3. Our model consists of five intermediate Holliday junction complexes describing the inversion/excision of the DNA sequence. FlpO dissociates from the resultant composite attachment sites to give free, genetically differentiated DNA. The excision reaction is not symmetrical, due to the spatial disparity of its products. Again, two FlpO monomers bound at each corresponding FRT site is sufficient to facilitate the excision event, and Holliday junction formation is identical to that of the inversion reaction. FlpO dissociation produces free disparate genetic products: the excised sequence of DNA whose exposed ends are ligated to form a loop, and the shortened DNA strand that remains. Transition to prior genetic states is made possible by the reversibility of the molecular reactions, however, spatial localisation is required for maximum efficiency in the case of transitioning back to State 1 from State 2 (not relevant for the applications presented here).

Although this circuit resembles biological logic gate circuitry in the literature, it has just one input and its only desired function is to implement an efficient ON switch from State 1 to State 2. The ON switch is designed to allow the activation of the CAR T cells to be delayed until an appropriate time point, based on the requirements of individual patients. The OFF switch can be considered as a separate circuit in which the initial positions of the blank sequence and CAR gene are swapped, and thereby only State 1 provides CAR expression. The OFF switch is designed to provide controlled cessation of an initial ON state as an alternative to more traditional kill switches that terminate the therapy completely. Both the ON and OFF switches are induced in the same manner, through 4‐OHT activation of the FlpO recombinase which then performs the necessary DNA recombination.

Based on a detailed analysis of the underlying reactions, we developed a set of biochemical equations representing the molecular interaction network shown in Fig. 2 (see supplementary material, Table S1). Our mechanistic model is derived through the application of mass action kinetics to these biochemical equations. The result is a system of ordinary differential equations (ODEs) that describes the rate of change in concentration of each molecular entity with respect to time (see supplementary material, Table S2). Since we are interested in the dynamical response of the system over time and have time course data with which to validate the model, this modelling approach provides suitable approximations to the time‐dependent fluctuations in the concentration of model variables in the ON state (the percentage of cells ON). The deterministic output provides an approximation to the average of an ensemble of stochastic simulations without the increased computational workload necessary for such probabilistic approaches.

To simulate the time course concentration dynamics of the genetic states of interest, we compute the total concentration of the system in these states by summing all ODEs describing molecular entities in the same state. Hence, we can derive the following two equations that capture the time course evolution of State 1 and State 2:

(1) $$\frac{dS_{1}}{dt}=k_{3}\left(D_{tS_{1}}^{i_{1}}F_{4}+D_{tS_{2}}^{i_{2}}F_{4}\right)-k_{-3}\left(H_{5}^{i_{1}}+H_{5}^{i_{2}}\right),$$

(2) $$\begin{array}{rl} \frac{dS_{2}}{dt} & =k_{-3}\left(H_{5}^{i_{1}}+H_{5}^{i_{2}}\right)-k_{3}\left(D_{tS_{1}}^{i_{1}}F_{4}+D_{tS_{2}}^{i_{2}}F_{4}\right)\\ & \;+\frac{k_{8}}{2}\left(D_{S_{2}}^{e_{1}}F_{2}\cdot D_{S_{X}}^{e_{1}}F_{2}+D_{S_{2}}^{e_{2}}F_{2}\cdot D_{S_{X}}^{e_{2}}F_{2}\right)\\ & \;-\frac{k_{-8}}{2}\left(H_{5}^{e_{1}}+H_{5}^{e_{2}}\right), \end{array}$$

where $S_{1}$, $tS_{1}$, $S_{2}$, $tS_{2}$ and $S_{X}$ denote State 1, transient State 1, State 2, transient State 2 and the deleted DNA state respectively; *D, H* and *F* denote DNA, Holliday junction complexes and FlpO, respectively; $i_{1}$, $i_{2}$ and $e_{1}$, $e_{2}$ denote each of the two inversion and excision reactions, respectively. Numerical subscripts for *H* and *F* denote the relevant Holliday junction complex (out of five) and the number of monomers, respectively. The numerical solutions to (1) and (2) constitute the outputs of our model simulations. The factor of half in the latter two terms in (2) is important for preserving the conservation of concentration (mass) in the model. A description of how this factor arises mathematically, using a simplified example, is provided in Supplementary Material, Section S1.

### 2.2 Model accurately predicts circuit switching efficiency and dynamics

Our experimental data on the performance of the inversion–excision switch consists of four time‐course datasets that record the percentage CAR expression of both the ON switch and OFF switch over a ten‐day period subject to 0 and 1 μM 4‐OHT dosages (see Section 3).

We employed two separate methods to fit the model to our time course data, due to the lack of experimental data regarding the values of our model parameters. The first method employs global optimisation using a genetic algorithm (GA). The GA mimics natural selection by evolving an initial population of randomly generated solutions over a large number of generations until it converges to a near global minimum within the allocated parameter space (see Section 3). Such algorithms have been widely used in mathematical model inference research relating to, e.g. synthetic oscillators and gene regulatory networks [48, 49, 50].

The second data fitting method is a statistical approach known as approximate Bayesian computation (ABC). This is implemented using ABC‐SysBio, a Python software package designed specifically for statistical parameter inference in biological systems research [51, 52, 53]. The programme employs sequential Monte Carlo (SMC) simulations to construct an accurate approximation to the posterior probability distribution defined by Bayes’ theorem:

$$P\left(A|B\right)=\frac{P\left(B|A\right)P\left(A\right)}{P\left(B\right)},\;P\left(B\right)>0.$$

Monte Carlo approaches involve computational simulations to generate random candidate solutions, testing their fitness against the desired output and repeating until a viable solution can be identified (see Section 3). ABC establishes distributions on each parameter value in order to infer the parameterisation most likely to have produced the data. In contrast, the GA identifies a single model parameterisation that provides minimal error between the associated in silico simulation and experimental data. However, a single parameterisation provides no information on the extent of uncertainty in the optimal values and is thus more susceptible to overfitting the data. Consequently, we seek maximum reliability in our results by initially performing global optimisation, then verifying this result by examining the likelihood of the existence of better solutions using ABC.

**Fig. 3:**
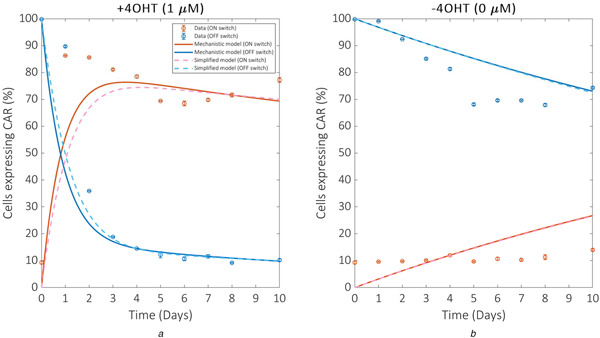
Data fitting results. The model is optimised against four time course datasets simultaneously: the ON and OFF switch responses to ***(a)*** +4‐OHT (1 μM 4‐OHT) and **(b)** −4‐OHT (0 μM 4‐OHT). Red and blue lines depict ON and OFF switch model simulations corresponding to red and blue circles depicting ON and OFF switch experimental data, respectively. Solid lines depict mechanistic model simulations. Dashed lines depict simplified model simulations

Global optimisation data fitting results confirm that the mechanistic model is capable of replicating time course percentage CAR expression for both ON and OFF switches over the ten‐day period recorded experimentally (Fig. 3, solid lines). The optimal parameter values identified by the GA are listed in supplementary material, Table S3. The error function used to assess the fitness of each solution calculates the absolute error across all four datasets. The optimal parameterisation provides an error of 28.15, which enables us to inform the selection of the sequence of error thresholds, $\epsilon_{i}$, for the subsequent ABC parameter inference. In this case, we set the penultimate error threshold, $\epsilon_{9},$ at 29 and the final threshold, $\epsilon_{10}$, at 28.15 under the assumption that solutions providing an error below 29 are sufficiently similar to our global optimal solution, and that convergence to the final threshold will present solutions superior to that of the GA. ABC parameter inference results reveal that a population of 100 parameterisations met the penultimate error threshold, but that convergence to the final threshold did not occur within a feasible time frame (Table 1). This provides strong evidence that the parameterisation identified by the GA is indeed near the global optimal solution since we are unable to statistically verify the existence of other solutions capable of providing a smaller error. Furthermore, our ABC approach provides distributions of each parameter value for accepted solutions at each error threshold. These distributions are non‐uniform and hence it is unlikely that multiple distinct parameterisations are capable of providing the same optimal model output. This also suggests that our model is identifiable; at least within a finite region of the parameter space.

**Table 1:** ABC‐SysBio parameter inference results

Error thresholds	Particles assessed	Particles accepted, %	Time, s
*ɛ*_1_ = 500	100	100.00	227
*ɛ*_2_ = 250	100	100.00	236
*ɛ*_3_ = 100	647	15.46	1674
*ɛ*_4_ = 50	2019	4.95	9031
*ɛ*_5_ = 45	2736	3.65	12,040
*ɛ*_6_ = 40	3873	2.58	16,821
*ɛ*_7_ = 35	8755	1.14	38,438
*ɛ*_8_ = 30	57,151	0.17	248,534
*ɛ*_9_ = 29	104,289	0.096	456,383
*ɛ*_10_ = 28.15	—	—	—

In order to clarify the key mechanistic features of the system, we conducted a sensitivity analysis to identify whether, and how, the model could be simplified without compromising performance (supplementary material, Fig. S1). Parameters are varied by up to two orders of magnitude above and below their optimal values. Sensitivity is examined with respect to response time, which is measured as the time taken for the ON switch to reach 90% of its maximum percentage CAR expression, and the time taken for the OFF switch to reach 90% of its total decrease in percentage CAR expression.

**Fig. 4:**
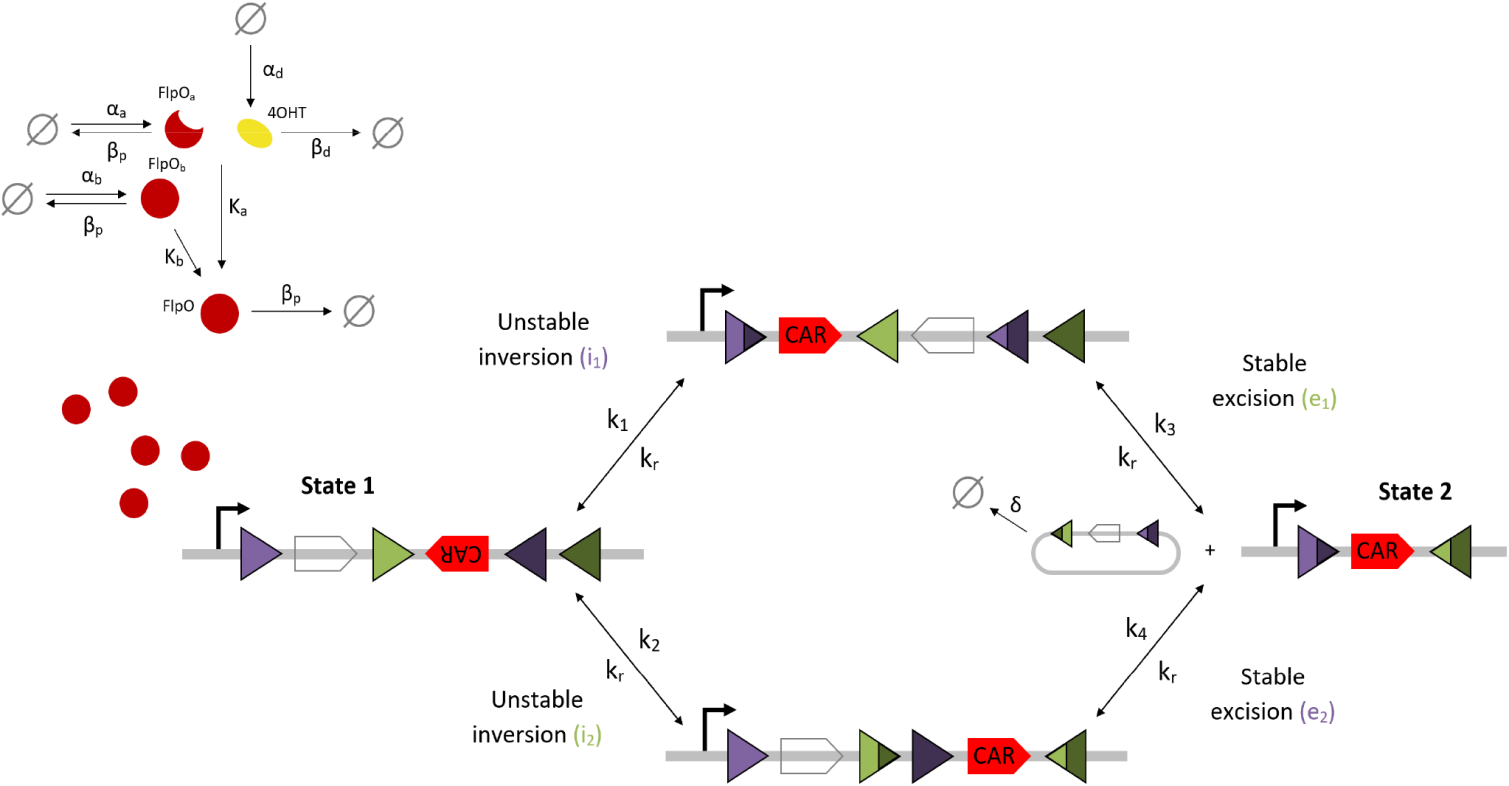
Schematic diagram of the reduced tyrosine recombinase‐based inversion–excision switch. FlpO recombinase, in both activated (FlpO) and inactivated (FlpO_a_, FlpO_b_) forms, targets gated FRT sites (purple and green triangles) in transitioning the switch from State 1 to State 2 via two transient intermediate genetic states. Clear and red pentagons depict the blank sequence and CAR gene respectively. Black arrows depict DNA:protein binding reactions comprising unstable inversion and stable excision events. Reaction rate constants are denoted by the corresponding numbered k. The parameter k_r_ denotes the rate of the reverse reaction for each recombination which enables further reduction of the number of model parameters

All parameters describing FlpO expression/degradation mechanisms within the system (*α_a_, α_b_, β_p_, β_d_, K_a_, K_b_*) showed high sensitivity, and are therefore retained in the simplified model. The parameters describing Holliday junction formation (*k*_4_, *k*_−4_, *k*_5_, *k*_−5_, *k*_6_, *k*_−6_, *k*_7_, *k*_−7_) showed relatively low sensitivity and are removed. The parameters describing cooperative FlpO monomer binding showed a high sensitivity for one pair of attachment sites (*i*_2_, *e*_1_: *k*_1_, *k*_−1_, *k*_2_, *k*_−2_), but comparatively low sensitivity for the other pair (*i*_1_, *e*_2_: *k*_1_, *k*_−1_, *k*_2_, *k*_−2_). Since each of the two pathways from State 1 to State 2 features recombination events involving each pair of attachment sites, we remove cooperative monomer binding and describe each recombination event as a single reversible reaction. The reduced model retains the four state variables relating to FlpO expression, activation, and degradation in the mechanistic model, but consists of just five DNA state variables compared to 63, while the number of parameters is reduced from 27 to 12 (Fig. 4). Applying mass action kinetics to the biochemical reactions depicted in Fig. 4 gives the following system of nine ODEs:

(3) $$\begin{array}{rl} \frac{d\left[F\right]}{dt} & =K_{a}\bar{V}\left[4OHT\right]\left[F_{a}\right]+K_{b}\bar{V}\left[F_{b}\right]-\beta_{p}\left[F\right]-k_{1}\left[F\right]\left[D_{S_{1}}\right]\\ & \;-k_{r}\left[F\right]\left[D_{tS_{1}}\right]-k_{2}\left[F\right]\left[D_{S_{1}}\right]-k_{r}\left[F\right]\left[D_{tS_{2}}\right]\\ & \;-k_{3}\left[F\right]\left[D_{tS_{1}}\right]-k_{4}\left[F\right]\left[D_{tS_{2}}\right], \end{array}$$

(4) $$\frac{d\left[4OHT\right]}{dt}=\alpha_{d}-\beta_{d}\left[4OHT\right]-K_{a}\left[4OHT\right]\left[F_{a}\right],$$

(5) $$\frac{d\left[F_{a}\right]}{dt}=\alpha_{a}-\beta_{p}\left[F_{a}\right]-K_{a}\left[4OHT\right]\left[F_{a}\right],$$

(6) $$\frac{d\left[F_{b}\right]}{dt}=\alpha_{b}-\beta_{p}\left[F_{b}\right]-K_{b}\left[F_{b}\right],$$

(7) $$\begin{array}{rl} \frac{d\left[D_{S_{1}}\right]}{dt} & =k_{r}\left[F\right]\left[D_{tS_{1}}\right]-k_{1}\left[F\right]\left[D_{S_{1}}\right]+k_{r}\left[F\right]\left[D_{tS_{2}}\right]\\ & \;-k_{2}\left[F\right]\left[D_{S_{1}}\right], \end{array}$$

(8) $$\begin{array}{rl} \frac{d\left[D_{tS_{1}}\right]}{dt} & =k_{1}\left[F\right]\left[D_{S_{1}}\right]-k_{r}\left[F\right]\left[D_{tS_{1}}\right]-k_{3}\left[F\right]\left[D_{tS_{1}}\right]\\ & \;+k_{r}\left[D_{S_{X}}\right]\left[D_{S_{2}}\right], \end{array}$$

(9) $$\begin{array}{rl} \frac{d\left[D_{tS_{2}}\right]}{dt} & =k_{2}\left[F\right]\left[D_{S_{1}}\right]-k_{r}\left[F\right]\left[D_{tS_{2}}\right]-k_{4}\left[F\right]\left[D_{tS_{2}}\right]\\ & \;+k_{r}\left[D_{S_{X}}\right]\left[D_{S_{2}}\right], \end{array}$$

(10) $$\begin{array}{rl} \frac{d\left[D_{S_{X}}\right]}{dt} & =k_{3}\left[F\right]\left[D_{tS_{1}}\right]-k_{r}\left[D_{S_{X}}\right]\left[D_{S_{2}}\right]+k_{4}\left[F\right]\left[D_{tS_{2}}\right]\\ & \;-k_{r}\left[D_{S_{X}}\right]\left[D_{S_{2}}\right]-\delta \left[D_{S_{X}}\right], \end{array}$$

Each population of accepted particles is formed by assessing the error provided by each particle. If the error is less than the threshold for that population the particle is accepted if not, the particle is rejected. Our inference required that 100 particles be accepted before progression to the next population and error threshold. The final error threshold, *ɛ*_10_, was chosen based on our GA optimisation result and ABC‐SysBio was unable to identify a population of particles that provide less error than this result

(11) $$\begin{array}{rl} \frac{d\left[D_{S_{2}}\right]}{dt} & =k_{3}\left[F\right]\left[D_{tS_{1}}\right]-k_{r}\left[D_{S_{X}}\right]\left[D_{S_{2}}\right]+k_{4}\left[F\right]\left[D_{tS_{2}}\right]\\ & \;-k_{r}\left[D_{S_{X}}\right]\left[D_{S_{2}}\right]. \end{array}$$

To assess the performance of the simplified model in reproducing the experimental data we again implemented global optimisation. GA data fitting results reveal that the simplified model is capable of matching the minimal error provided by the optimal mechanistic model parameterisation (Fig. 3, dashed lines). The optimal parameterisation for the reduced model provides an error of 28.39, a negligible increase on the 28.15 provided by the mechanistic model. The optimal parameter values for the reduced model are listed in supplementary material, Table S4. Further attempts to simplify the model by removing other features resulted in large increases in the fitting error, providing strong evidence that this model represents the simplest possible mechanistic representation of the system. All results described hereafter were derived from this simplified model of the system.

### 2.3 Switching response times are inversely proportional to the concentration of inducer drug

**Fig. 5:**
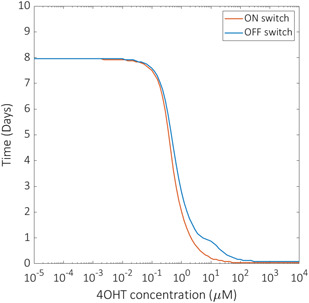
Model predictions of drug dosage dependency. Optimised reduced model simulations of response time as a function of 4‐OHT concentration for both the ON switch (red line) and OFF switch (blue line). Response time is measured as the time taken for the ON switch to reach 90% of its maximum percentage CAR expression, and the time taken for the OFF switch to reach 90% of its total decrease in percentage CAR expression

Response time is the key performance characteristic of the switch since ultimately it will need to function reliably in mammalian cells within time frames that are appropriate to the condition of individual patients. By identifying parameters that are both influential over system output and experimentally tuneable, we can establish guidelines to optimise the functioning of the switch in different application scenarios. Model simulations reveal that there is an inverse proportionality between drug concentration and switching response time (Fig. 5). The response time of both switches is significantly decreased when induced by 4‐OHT concentrations in the range 10^−1^–10^1^ μM. Therefore the 1 μM 4‐OHT concentration used experimentally may require an increase of up to 10 μM to deliver improved response times. The concentration of 4‐OHT used to induce the switch is important as it has implications regarding the cost of the treatment. Identifying key operational factors through model simulations ultimately informs the direction of confirmatory experimental work and presents new avenues of investigation in a time efficient manner. Given that the treatment is likely to benefit from minimising the concentration of the inducer drug, the model can be utilised to identify alternative parameters that have the potential to improve switch response time.

### 2.4 Increasing Flp0 expression can provide faster switching

**Fig. 6:**
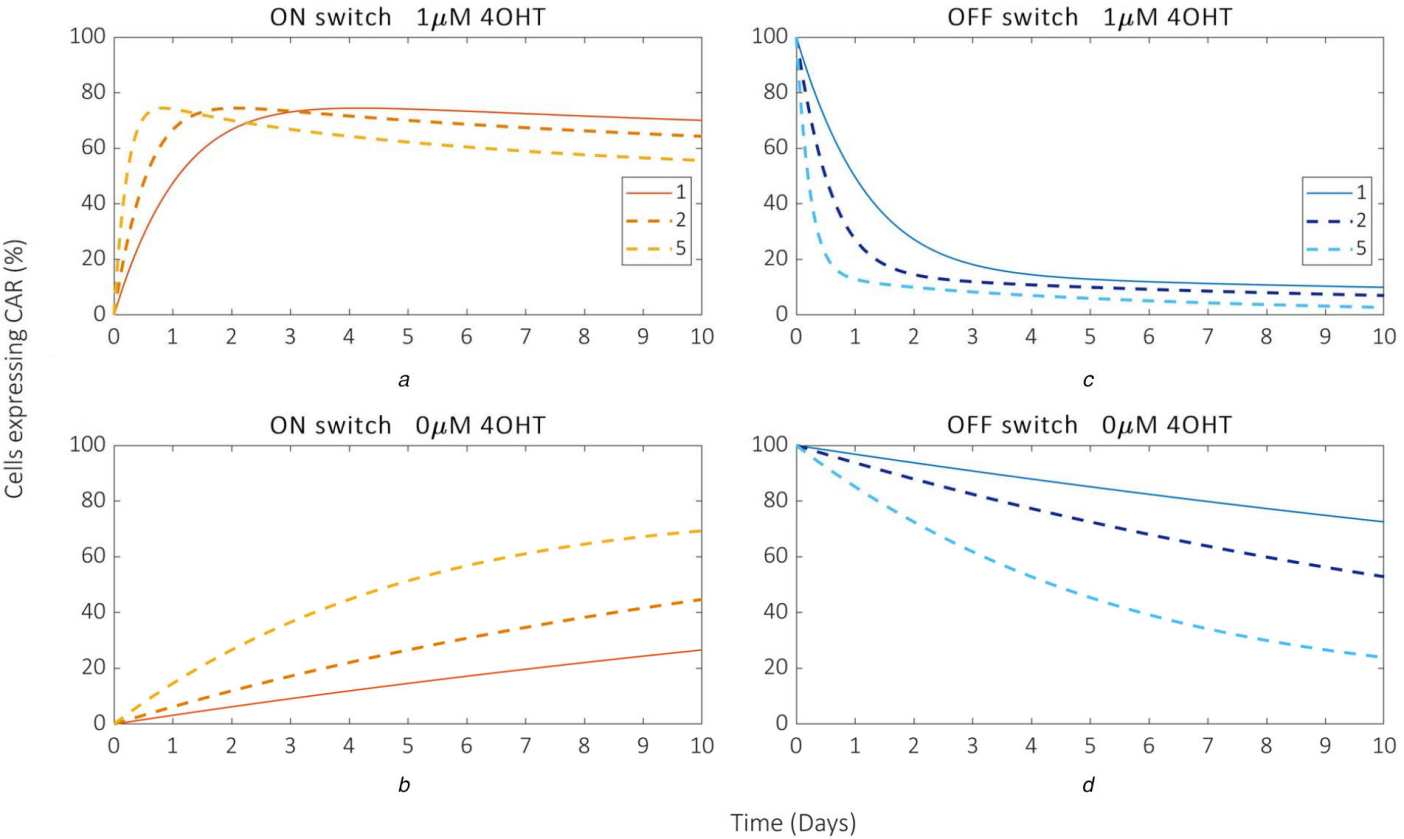
Model predictions of the effect of tuning FlpO expression on switch response times. Model simulations of ON switch and OFF switch time course responses for the optimal values of α_a_ and α_b_ subject to fold changes of one, two and five **(*a*)** ON switch + 4‐OHT (1 μM), **(*b*)** ON switch − 4‐OHT (0 μM), **(*c*)** OFF switch + 4‐OHT (1 μM), **(*d*)** OFF switch − 4‐OHT (0 μM)

Sensitivity analysis of the reduced model parameters (supplementary material, Fig. S2) identifies the most sensitive to be *α*_a_, *β*_p_, *β*_d_, and *K*_a_. Of these, the rates of protein and drug degradation, *β*_p_ and *β*_d_, and the rate of FlpO nuclear localisation through 4‐OHT activation, *K*_a_, are challenging candidates for experimental tuning. However, the rate of FlpO expression in the system, *α_a_* and *α_b_* is potentially tuneable through appropriate choice of the cognate promoter strength. Model simulations reveal faster response times for both the ON and OFF switches as the values of *α*_a_ and *α*_b_ are increased two‐ and five‐fold (Fig. 6), with a significant reduction in switch response time for even a two‐fold increase in FlpO expression. Recent work on engineering Jurkat T cells with inducible promoters of varying strengths illustrates the potential to provide the required increase in FlpO expression. However, the model predicts that increased FlpO expression also results in a decreased percentage of CAR expression over time with respect to the ON switch (Figs. 6*a* and *b*). For example, a five‐fold increase in FlpO expression is predicted to provide >75% cells expressing CAR within a relatively small time frame (∼1 day), but this reduces to ∼55% after 2 days. Therefore, unless the action of the CAR T cells is as effective as to provide significant therapeutic benefit over small time periods, these dynamics are unlikely to be advantageous. By contrast, the performance of the OFF switch improves uniformly as FlpO expression increases.

The model also predicts that increased FlpO expression causes increased basal switching responses from both switches (Figs. 6*c* and *d*) that could potentially compromise the efficacy of the therapy altogether, regardless of which switch was considered to be most viable. Hence, attempts to improve the efficiency of the switch through increased FlpO expression would not be suitable for applications in which tight regulation of the initial state in the absence of induction is required.

### 2.5 Optimal switch performance presents a trade‐off between 4‐OHT concentration and FlpO expression

**Fig. 7:**
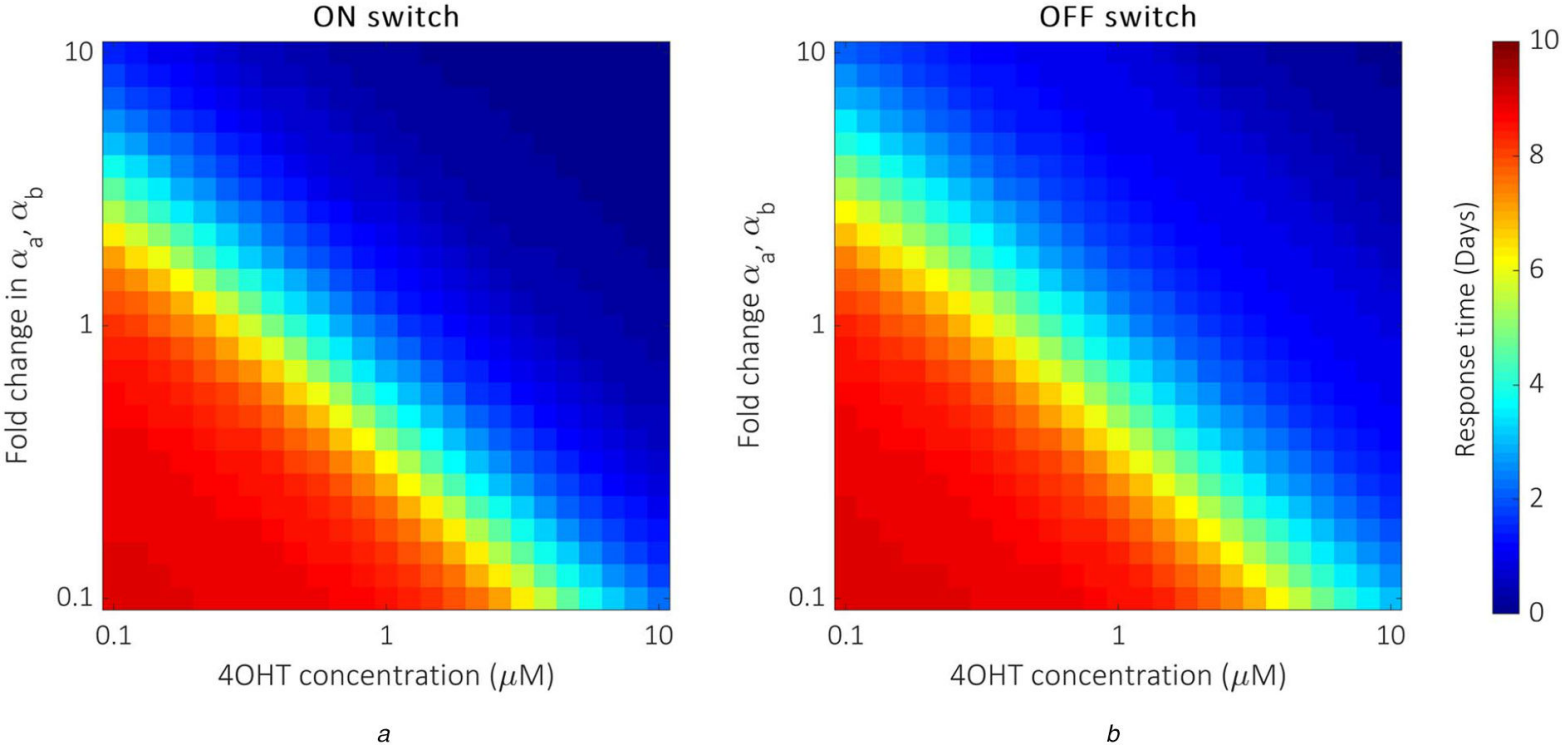
Model predictions of parameter tuning trade‐offs **(*a*)** Model simulations of ON switch response time for the optimal values of *α_a_* and *α_b_* subject to fold changes of one order of magnitude above and below the optimal values, and for 4‐OHT concentrations between 10^−1^ and 10^1^ μM, **(*b*)** Model simulations of OFF switch response time for the optimal values of *α_a_* and *α_b_* subject to fold changes of one order of magnitude above and below the optimal values, and for 4‐OHT concentrations between 10^−1^ and 10^1^ μM. Response time is measured as the time taken for the ON switch to reach 90% of its maximum percentage CAR expression, and the time taken for the OFF switch to reach 90% of its total decrease in percentage CAR expression. The colour gradient ranges from dark blue (0 days) to dark red (10 days)

Investigating the effects of simultaneously modifying the expression of FlpO and the concentration of 4‐OHT reveals that improved switch response times are possible for relatively large doses of 4‐OHT if FlpO expression is unaltered, and for relatively low doses of 4‐OHT if FlpO expression is increased ∼10‐fold (Fig. 7). The model predicts that increasing FlpO expression ∼2‐fold and using a 4‐OHT dosage of ∼3 μM can provide a notable improvement in response times for both the ON and OFF switches. Although this demonstrates an advantageous reduction in the magnitude of the required fold change in FlpO expression for a relatively small increase in drug dosage, the resulting basal activity increases to potentially problematic levels, suggesting that tuning inducer concentration/dosage may be a more viable method for improving switch efficiency.

## 3 Materials and methods

### 3.1 Experimental procedure

Primary CD4 + T cells were harvested from blood obtained from the Boston Children's Hospital using the STEMCELL CD4 + enriched cocktail and RosetteSep system. T cells were maintained in X‐VIVO 15 media (Lonza) supplemented with 5% AB Serum (Valley Biomedical), 10 mM *N*‐acetyl L‐cystein (Sigma), 55 µM 2‐mercaptoethanol (Gibco), and 50–100 units/ml recombinant interleukin‐2 (IL‐2) (Tecin, NCI BRB Preclinical Repository).

Lentivirus containing either the inducible FlpO or stable inversion switch was produced via polyethylenimine transfection of the human embryonic kidney 293 FT cells and collected 3 and 4 days post‐transfection. The concentrated virus was produced via ultracentrifugation with 20% sucrose (Sigma) for 2 h at 4°C and 22,000 *g*. T cells were activated with CD3/CD28 Dynabeads (Gibco), and then spinfected with a virus one day later. Using half of the concentrated virus, both inducible recombinase and stable inversion switch viruses were spun onto the well a six‐well retronectin (Clontech)‐coated plate for 90 min at 1200 *g* and activated CD4 + T cells were then spun on to the virus plates for 60 min at 1200 *g*. T cells were sorted using the SH800 Cell Sorter (Sony, BFP‐FL1 channel) for FlpOER^T2^ expression using a BFP marker expressed in the FlpOER^T2^ cassette via a T2a ribosomal skip sequence.

Cells were induced with 1 µM 4‐hydroxytamoxifen (4‐OHT, Sigma) in methanol solution except for dose response experiments. Cells were induced at a starting concentration of 200,000 cells/ml and maintained between 200,000–1200,000 cells/ml with media containing 4‐OHT. Uninduced cells were also plated and maintained at the same cell concentrations in inducer‐free media. CAR expression was observed via phycoerythrin (PE)‐conjugated antibody staining (R&D Systems IC3696P) for a human myc epitope tag expressed in the extracellular portion of the CAR, which was then quantified via flow cytometry (Attune NxT Flow Cytometer).

The percentage switching efficiency is thought to reflect the efficiency of integrating both the FlpO‐producing component and the switch itself into the T cells. Given that successful integration is not guaranteed for every cell, there are four possible outcomes: R−/FS− (no components), R+/FS− (recombinase only), R−/FS+ (switch only) and R+/FS+ (recombinase and switch). Only cells that have acquired the requisite components (R+/FS+) can provide the desired function, and hence we convert our data to represent percentage CAR expression with respect to the global maximum data value to focus our analysis purely on the R+/FS+ cells in the population. We assume that integration efficiency does not vary significantly across experimental trials, allowing us to use the global maximum data value as a metric for functionally viable T cell populations.

All cells are thought to possess an initial volumetric ratio (the ratio of the volume of the cytoplasm to the volume of the nucleus, $V_{C}:V_{N}$) of ∼4:1 that changes with cell growth, becoming 2:1 or 1:1. However, lymphocytes such as T cells have not been found to demonstrate these growth related adaptations, maintaining a ratio between 4:1 and 3:1. Hence, the parameter $\bar{V}$, equal to the quotient $V_{C}/V_{N}$, is fixed at 0.3 in our model.

We imposed the conditions $k_{1}<k_{2}$ and $k_{-1}>k_{-2}$ to account for cooperative FlpO monomeric binding to DNA, whereby the binding affinity of a FlpO monomer to an attachment site already bound by another monomer is greater than that of a monomer to a free site. This involves setting $k_{1}=k_{2}$ and $k_{-1}=k_{-2}$ and introducing the multipliers $0\;<m_{1}<1$ and $0<m_{2}<1$ such that $m_{1}$ multiplies $k_{1}$ and $m_{2}$ multiplies $k_{-2}$, hence ensuring that $k_{1}/k_{-1}<k_{2}/k_{-2}$ in our parameter inference.

### 3.2 Model parameter inference and simulation

#### 3.2.1 Global optimisation

We employed the built‐in GA function in MATLAB for parameter inference purposes. The GA proceeds by initially generating a population of solutions at random within the predefined parameter space. The initial solutions are then scored based on their fitness. This is typically calculated by virtue of an error function that determines how well each solution is able to match the relevant experimental data. The best solutions are selected as parents that will produce the best offspring to populate the next generation of solutions. Parents produce offspring through crossover, whereby a random place in their binary genotype is selected and the information beyond that point is swapped over. The mutation is also incorporated, whereby single point alterations in the offspring's genotype are imposed to increase diversity within the population. This procedure is repeated indefinitely until a predefined termination criterion is reached; typically, the scores are unchanged over a specified number of generations, the algorithm reaches a specified number of generations, or a specified solution score is established. Parallelisation of the MATLAB code enabled us to run the GA with a large search population for many generations; this significantly increases the likelihood of establishing the global optimal solution. Given the lack of available data regarding the relevant reaction rate constants in the literature, we require a parameter space large enough to locate optimal solutions, but not so big that convergence timescales become impractical. A large parameter space increases the time taken to produce the next generation of solutions however, this can also decrease the overall number of generations required for convergence, since more solutions are inspected in each case. Large parameter spaces also increase the risk of incurring problems with stiff model simulations that may cause the GA to fail, hence multiple trials are often required to determine effective performance criteria. Hence, the search interval imposed on all model parameters is [0, 1]. We ran the GA over 1000 generations with a population size of at least 1000 in order to maximise the likelihood of convergence and the identification of the global optimum solution. We define the error function that calculates the mean absolute error between the four time‐course datasets and their corresponding model simulations such that

$$d=\frac{1}{10}\sum_{i=1}^{40}\left|x_{i}-y_{i}\right|,$$

where $x_{i}$ and $y_{i}$ are corresponding elements of the experimental data and model simulation vectors, respectively. Each of the four time‐course datasets consists of ten values and our distance function calculates the mean absolute error across all four datasets.

#### 3.2.2 Approximate Bayesian computation (ABC)

The ABC–SMC procedure implemented by ABC‐SysBio proceeds in the following manner: an initial population of solutions (particles) is randomly generated in accordance with the prior distributions imposed on the model parameters. Each particle provides a simulated dataset, $D^{⋆}$, which is compared to the fixed experimental dataset, *D*, by an appropriate distance (error) function and its fitness is scored accordingly. This error score determines the acceptance of a particle, dependent on a decreasing sequence of error thresholds, ϵ, set to correspond with each population, i.e.

$$d\left(D^{⋆},D\right)<\epsilon_{i},$$

where ε_1_ > ε_2_ > … > ε*_n_* and *d* is the distance function. We define our distance function as the mean absolute error between model simulations and experimental data in the same way as the GA approach. Subsequent populations are obtained by perturbing particles from the previous population in accordance with a predetermined perturbation kernel, proceeding until the model is unable to produce particles of sufficient fitness to satisfy the immediate threshold.

An array of model‐specific criteria are required to allow the ABC‐SysBio package to run efficiently: the sequence of decreasing error thresholds, ϵ, must be provided whereby only the particles capable of providing error less than that of the threshold will be accepted by the algorithm. Each ϵ must be satisfied in succession until the particles are unable to satisfy the next threshold. The satisfaction of an individual threshold is dependent on the number of particles accepted; the number of acceptable particles required before passage to the next threshold must also be predetermined. The larger the number of particles, the higher probability of reliable inference results and the longer the time taken by the algorithm to converge. Each individual parameter subject to inference requires a prior probability distribution in order to establish the parameter space within which to locate acceptable particles. We allocate the same parameter space used in GA parameter inference to facilitate direct comparisons of the results. Hence, the prior distributions imposed on all model parameters are uniform distributions on the interval [0, 1]. The convergence of the algorithm is dependent on all of the aforementioned factors and hence it may require several trials to establish the appropriate performance criteria. To achieve credible results, it is advised that parameter inference and model selection tasks are repeated multiple times due to the random nature of the Monte Carlo simulations that drive the algorithm.

## 4 Conclusion

Mathematical models of synthetic circuits that are based strictly on underlying biological mechanisms can be used as design tools to reduce development times, lower the risk of circuit failure, and identify key parameters determining circuit performance. We have developed the first mechanistic mathematical model of a tyrosine recombinase‐based inversion–excision switch and validated it against dynamic data on CAR expression using both global optimisation and statistical approaches. A sensitivity analysis of this model revealed key mechanistic features that allowed the construction of a significantly simplified model that yielded equivalent data fitting results. Analysis of this model revealed that the response time of the switch can be modulated experimentally by tuning the concentration of the inducer drug and/or by choosing promoters that increase the level of expression of the FlpO recombinase. However, both of these solutions also have potential drawbacks in terms of increasing basal expression activity, indicating that the successful clinical deployment of this and other novel recombinase‐based circuits might be dependent on the development of new mechanisms for minimising or even nullifying basal recombinase expression.

## Supporting information

Supporting Information
