# Supplementary material for: Mechanistic modelling of tyrosine recombination reveals key parameters determining the performance of a CAR T cell switching circuit
Source: Eng Biol. 2020 Mar 24;4(1):10–9. doi: 10.1049/enb.2019.0020 (PMC9996713; doi:10.1049/enb.2019.0020)
Supplement: Supplementary file 1 — Supporting Information [file ENB2-4-10-s001.pdf]

Figure S1: Sensitivity analysis (mechanistic model)

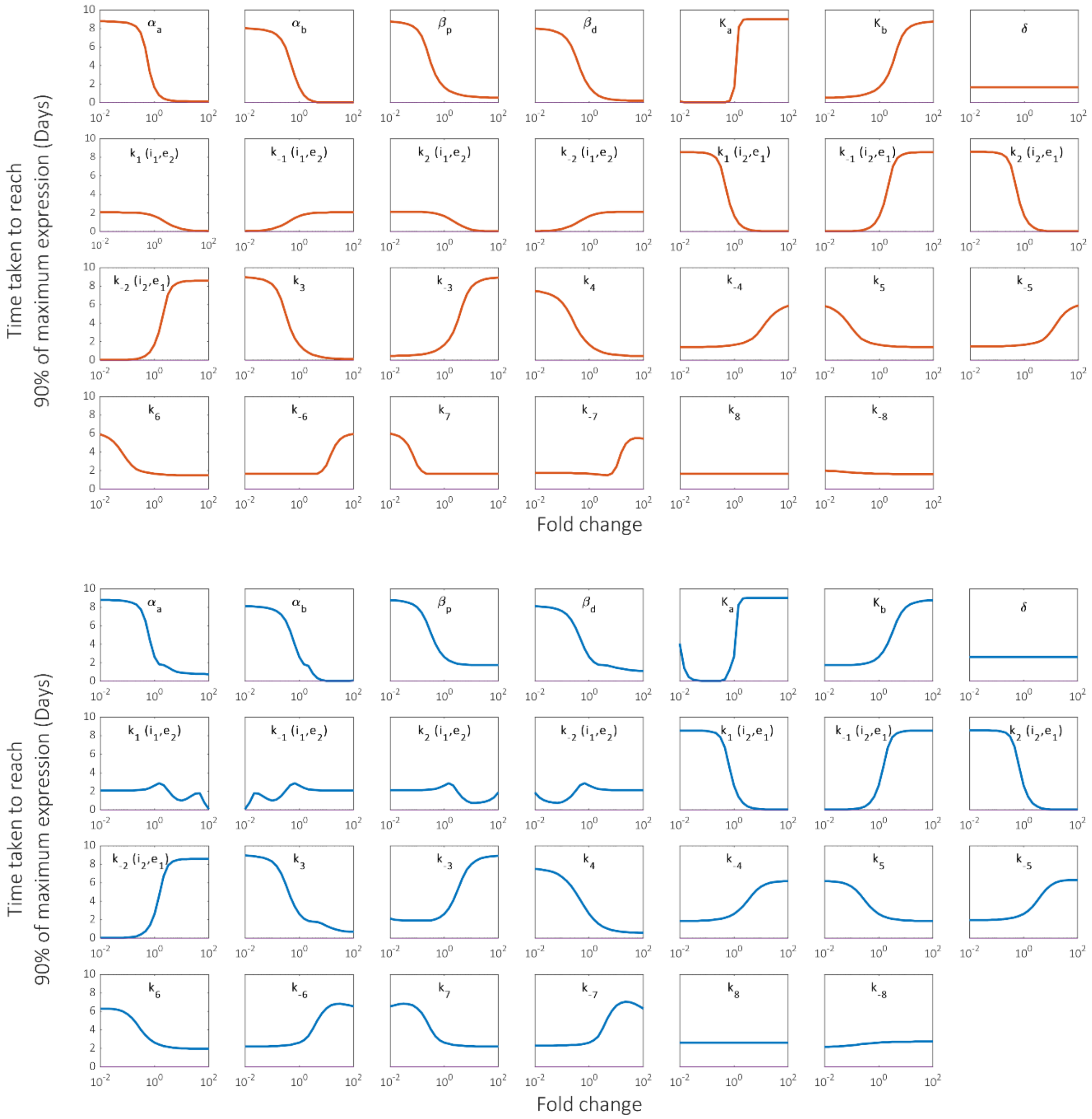

Figure 1: Mechanistic model parameters are varied by two orders of magnitude above and below their optimal values to examine their sensitivity with respect to the response time of the ON switch (red lines) and OFF switch (blue lines). Response time is measured as the time taken for the ON switch to reach 90% of its maximum percentage CAR expression, and the time taken for the OFF switch to reach 90% of its total decrease in percentage CAR expression. In all simulations the concentration of 4OHT is fixed at 1 μM.

Figure S2: Sensitivity analysis (reduced model)

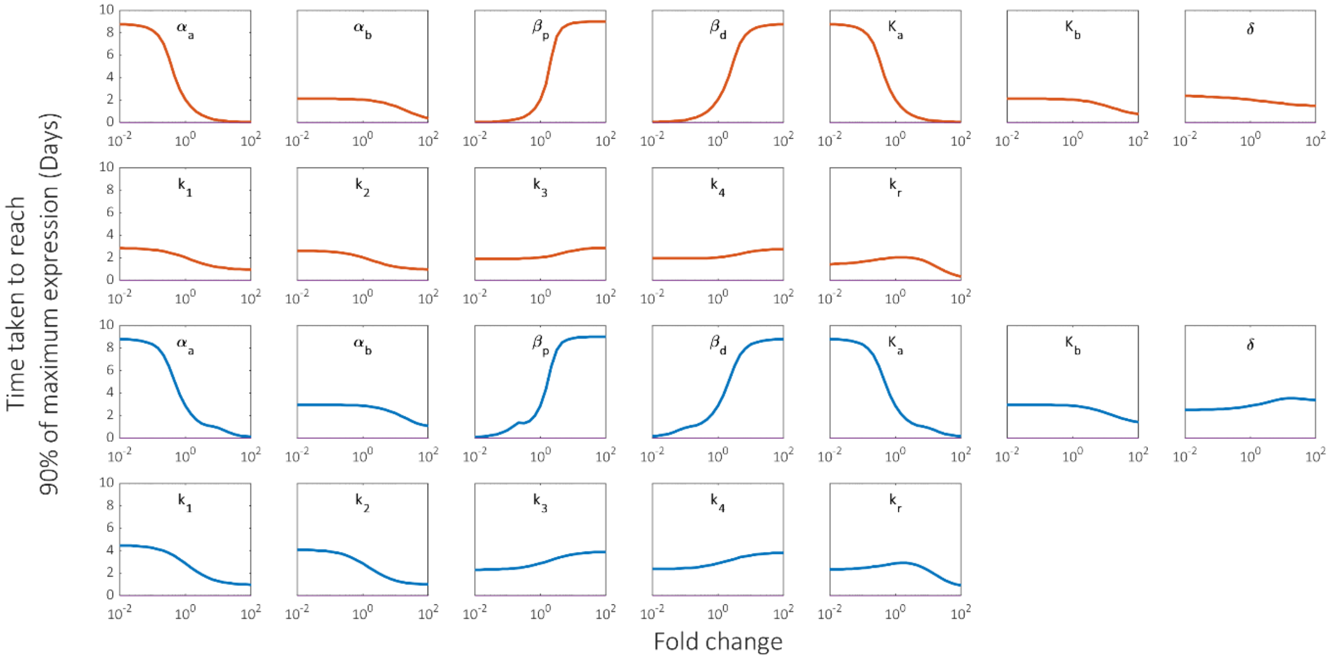

Figure 2: Reduced model parameters are varied by two orders of magnitude above and below their optimal values to examine their sensitivity with respect to the response time of the ON switch (red lines) and OFF switch (blue lines). Response time is measured as the time taken for the ON switch to reach 90% of its maximum percentage CAR expression, and the time taken for the OFF switch to reach 90% of its total decrease in percentage CAR expression. In all simulations the concentration of 4OHT is fixed at 1 μM.

Table S1: Biochemical equations

$$\varnothing \xrightleftharpoons[\beta_p]{\alpha_a} F_a$$
$$\varnothing \xrightleftharpoons[\beta_p]{\alpha_b} F_b$$
$$\varnothing \xrightleftharpoons[\beta_d]{\alpha_d} 4OHT$$
$$F_a + 4OHT \xrightarrow{K_a} F$$
$$F_b \xrightarrow{K_b} F$$
$$F \xrightarrow{\beta_p} \varnothing$$

$$D_{S_1} + F \xrightleftharpoons[k_{-1}]{k_1} D_{S_1}^{i_1,i_2}F$$
$$D_{S_1}^{i_1,i_2}F + F \xrightleftharpoons[k_{-2}]{k_2} D_{S_1}^{i_1,i_2}F_{2,0}$$
$$D_{S_1}^{i_1,i_2}F + F \xrightleftharpoons[k_{-1}]{k_1} D_{S_1}^{i_1,i_2}F_{1,1}$$
$$D_{S_1}^{i_1,i_2}F_{2,0} + F \xrightleftharpoons[k_{-1}]{k_1} D_{S_1}^{i_1,i_2}F_3$$
$$D_{S_1}^{i_1,i_2}F_{1,1} + F \xrightleftharpoons[k_{-2}]{k_2} D_{S_1}^{i_1,i_2}F_3$$
$$D_{S_1}^{i_1,i_2}F_3 + F \xrightleftharpoons[k_{-2}]{k_2} D_{S_1}^{i_1,i_2}F_4$$
$$D_{S_1}^{i_1,i_2}F_4 \xrightleftharpoons[k_{-3}]{k_3} H_1^{i_1,i_2}$$
$$H_1^{i_1,i_2} \xrightleftharpoons[k_{-4}]{k_4} H_2^{i_1,i_2}$$
$$H_2^{i_1,i_2} \xrightleftharpoons[k_{-5}]{k_5} H_3^{i_1,i_2}$$
$$H_3^{i_1,i_2} \xrightleftharpoons[k_{-6}]{k_6} H_4^{i_1,i_2}$$
$$H_4^{i_1,i_2} \xrightleftharpoons[k_{-7}]{k_7} H_5^{i_1,i_2}$$
$$D_{tS_2,tS_1}^{i_1,i_2}F_4 \xrightleftharpoons[k_{-3}]{k_3} H_5^{i_1,i_2}$$
$$D_{tS_2,tS_1}^{i_1,i_2}F_3 + F \xrightleftharpoons[k_{-2}]{k_2} D_{tS_2,tS_1}^{i_1,i_2}F_4$$
$$D_{tS_2,tS_1}^{i_1,i_2}F_{1,1} + F \xrightleftharpoons[k_{-2}]{k_2} D_{tS_2,tS_1}^{i_1,i_2}F_3$$
$$D_{tS_2,tS_1}^{i_1,i_2}F_{2,0} + F \xrightleftharpoons[k_{-1}]{k_1} D_{tS_2,tS_1}^{i_1,i_2}F_3$$
$$D_{tS_2,tS_1}^{i_1,i_2}F + F \xrightleftharpoons[k_{-1}]{k_1} D_{tS_2,tS_1}^{i_1,i_2}F_{1,1}$$
$$D_{tS_2,tS_1}^{i_1,i_2}F + F \xrightleftharpoons[k_{-2}]{k_2} D_{tS_2,tS_1}^{i_1,i_2}F_{2,0}$$
$$D_{tS_2,tS_1} + F \xrightleftharpoons[k_{-1}]{k_1} D_{tS_2,tS_1}^{i_1,i_2}F$$

$$D_{tS_2,tS_1} + F \xrightleftharpoons[k_{-1}]{k_1} D_{tS_2,tS_1}^{e_1,e_2}F$$
$$D_{tS_2,tS_1}^{e_1,e_2}F + F \xrightleftharpoons[k_{-2}]{k_2} D_{tS_2,tS_1}^{e_1,e_2}F_{2,0}$$
$$D_{tS_2,tS_1}^{e_1,e_2}F + F \xrightleftharpoons[k_{-1}]{k_1} D_{tS_2,tS_1}^{e_1,e_2}F_{1,1}$$
$$D_{tS_2,tS_1}^{e_1,e_2}F_{2,0} + F \xrightleftharpoons[k_{-1}]{k_1} D_{tS_2,tS_1}^{e_1,e_2}F_3$$
$$D_{tS_2,tS_1}^{e_1,e_2}F_{1,1} + F \xrightleftharpoons[k_{-2}]{k_2} D_{tS_2,tS_1}^{e_1,e_2}F_3$$
$$D_{tS_2,tS_1}^{e_1,e_2}F_3 + F \xrightleftharpoons[k_{-2}]{k_2} D_{tS_2,tS_1}^{e_1,e_2}F_4$$
$$D_{tS_2,tS_1}^{e_1,e_2}F_4 \xrightleftharpoons[k_{-3}]{k_3} H_1^{e_1,e_2}$$
$$H_1^{e_1,e_2} \xrightleftharpoons[k_{-4}]{k_4} H_2^{e_1,e_2}$$
$$H_2^{e_1,e_2} \xrightleftharpoons[k_{-5}]{k_5} H_3^{e_1,e_2}$$
$$H_3^{e_1,e_2} \xrightleftharpoons[k_{-6}]{k_6} H_4^{e_1,e_2}$$
$$H_4^{e_1,e_2} \xrightleftharpoons[k_{-7}]{k_7} H_5^{e_1,e_2}$$
$$D_{S_2}^{e_1,e_2}F_2 + D_{S_X}^{e_1,e_2}F_2 \xrightleftharpoons[k_{-3}]{k_3} H_5^{e_1,e_2}$$
$$D_{S_2}^{e_1,e_2}F + F \xrightleftharpoons[k_{-2}]{k_2} D_{S_2}^{e_1,e_2}F_2$$
$$D_{S_2} + F \xrightleftharpoons[k_{-1}]{k_1} D_{S_2}^{e_1,e_2}F$$
$$D_{S_X}^{e_1,e_2}F + F \xrightleftharpoons[k_{-2}]{k_2} D_{S_X}^{e_1,e_2}F_2$$
$$D_{S_X} + F \xrightleftharpoons[k_{-1}]{k_1} D_{S_X}^{e_1,e_2}F$$
$$D_{S_X} \xrightarrow{\delta} \varnothing$$

Table 1: Biochemical equations describing the full reaction network of the inversion-excision switch (Fig. S1). Reactions are paired for the two separate recombination events (inversion and excision) for the sake of brevity i.e. one set of equations for i_1 and i_2, and another set for e_1 and e_2. Activated FlpO that catalyses recombination events in the nucleus is denoted by F; unactivated FlpO in the cytosol is denoted by F_a; unactivated FlpO that leaks into the nucleus is denoted by F_b. FlpO monomers bind to DNA attachment sites sequentially until two monomers occupy each site; $F_{1,1}$ and $F_{2,0}$ denote one monomer bound to each site and two monomers bound to one site, respectively. DNA is denoted by D with a subscript corresponding to one of the five DNA states (S_1, tS_1, tS_2, S_2, S_X), and with a superscript corresponding to one of the four recombination events (i_1, i_2, e_1, e_2). Each of the five strand exchanges that comprise a Holliday junction is denoted by the corresponding numbered H.

Table S2: Model ODEs

$$
\begin{aligned}
\frac{d[F_a]}{dt} &= \alpha_a - K_a[F_a][4OHT] - \beta_p[F_a], \\
\frac{d[F_b]}{dt} &= \alpha_b - K_b[F_b] - \beta_p[F_b], \\
\frac{d[4OHT]}{dt} &= \alpha_d - K_a[F_a][4OHT] - \beta_d[4OHT], \\
\frac{d[F]}{dt} &= \bar{V}K_a[F_a][4OHT] + \bar{V}K_b[F_b] - \beta_p[F]+ \\
&\quad k_{-1}[D^{i1}_{tS_2}F] - k_1[D_{tS_2}][F] + k_{-2}[D^{i1}_{S_1}F_{2,0}] - k_2[D^{i1}_{S_1}F][F] + k_{-1}[D^{i1}_{S_1}F_{1,1}] - k_1[D^{i1}_{S_1}F][F] + k_{-1}[D^{i1}_{S_1}F_3] - k_1[D^{i1}_{S_1}F_{2,0}][F]+ \\
&\quad k_{-2}[D^{i1}_{S_1}F_3] - k_2[D^{i1}_{S_1}F_{1,1}][F] + k_{-2}[D^{i1}_{S_1}F_4] - k_2[D^{i1}_{S_1}F_3][F] + k_{-1}[D^{i2}_{S_1}F] - k_1[D_{S_1}][F] + k_{-2}[D^{i2}_{S_1}F_{2,0}] - k_2[D^{i2}_{S_1}F][F]+ \\
&\quad k_{-1}[D^{i2}_{S_1}F_{1,1}] - k_1[D^{i2}_{S_1}F][F] + k_{-1}[D^{i2}_{S_1}F_3] - k_1[D^{i2}_{S_1}F_{2,0}][F] + k_{-2}[D^{i2}_{S_1}F_3] - k_2[D^{i2}_{S_1}F_{1,1}][F] + k_{-2}[D^{i2}_{S_1}F_4] - k_2[D^{i2}_{S_1}F_3][F]+ \\
&\quad k_{-1}[D^{i1}_{S_1}F] - k_1[D_{S_1}][F] + k_{-2}[D^{i1}_{S_1}F_{2,0}] - k_2[D^{i1}_{S_1}F][F] + k_{-1}[D^{i1}_{S_1}F_{1,1}] - k_1[D^{i1}_{S_1}F][F] + k_{-1}[D^{i1}_{S_1}F_3] - k_1[D^{i1}_{S_1}F_{2,0}][F]+ \\
&\quad k_{-2}[D^{i1}_{S_1}F_3] - k_2[D^{i1}_{S_1}F_{1,1}][F] + k_{-2}[D^{i1}_{S_1}F_4] - k_2[D^{i1}_{S_1}F_3][F] + k_{-1}[D^{i2}_{S_1}F] - k_1[D_{S_1}][F] + k_{-2}[D^{i2}_{S_1}F_{2,0}] - k_2[D^{i2}_{S_1}F][F]+ \\
&\quad k_{-1}[D^{i2}_{S_1}F_{1,1}] - k_1[D^{i2}_{S_1}F][F] + k_{-1}[D^{i2}_{S_1}F_3] - k_1[D^{i2}_{S_1}F_{2,0}][F] + k_{-2}[D^{i2}_{S_1}F_3] - k_2[D^{i2}_{S_1}F_{1,1}][F] + k_{-2}[D^{i2}_{S_1}F_4] - k_2[D^{i2}_{S_1}F_3][F]+ \\
&\quad k_{-1}[D^{i2}_{tS_1}F] - k_1[D_{tS_1}][F] + k_{-2}[D^{i2}_{tS_1}F_{2,0}] - k_2[D^{i2}_{tS_1}F][F] + k_{-1}[D^{i2}_{tS_1}F_{1,1}] - k_1[D^{i2}_{tS_1}F][F] + k_{-1}[D^{i2}_{tS_1}F_3] - k_1[D^{i2}_{tS_1}F_{2,0}][F]+ \\
&\quad k_{-2}[D^{i2}_{tS_1}F_3] - k_2[D^{i2}_{tS_1}F_{1,1}][F] + k_{-2}[D^{i2}_{tS_1}F_4] - k_2[D^{i2}_{tS_1}F_3][F] + k_{-1}[D^{e2}_{tS_1}F] - k_1[D_{tS_1}][F] + k_{-2}[D^{e2}_{tS_1}F_{2,0}] - k_2[D^{e2}_{tS_1}F][F]+ \\
&\quad k_{-1}[D^{e2}_{tS_1}F_{1,1}] - k_1[D^{e2}_{tS_1}F][F] + k_{-1}[D^{e2}_{tS_1}F_3] - k_1[D^{e2}_{tS_1}F_{2,0}][F] + k_{-2}[D^{e2}_{tS_1}F_{1,1}] - k_2[D^{e2}_{tS_1}F][F] + k_{-2}[D^{e2}_{tS_1}F_4] - k_2[D^{e2}_{tS_1}F_3][F]+ \\
&\quad k_{-1}[D^{e1}_{S_2}F] - k_1[D_{S_2}][F] + k_{-2}[D^{e1}_{S_2}F_2] - k_2[D^{e1}_{S_2}F][F] + k_{-1}[D^{e2}_{S_2}F] - k_1[D_{S_2}][F] + k_{-2}[D^{e2}_{S_2}F_2] - k_2[D^{e2}_{S_2}F][F]+ \\
&\quad k_{-1}[D^{e1}_{S_X}F] - k_1[D_{S_X}][F] + k_{-2}[D^{e1}_{S_X}F_2] - k_2[D^{e1}_{S_X}F][F] + k_{-1}[D^{e2}_{S_X}F] - k_1[D_{S_X}][F] + k_{-2}[D^{e2}_{S_X}F_2] - k_2[D^{e2}_{S_X}F][F], \\
\frac{d[D_{tS_1}]}{dt} &= k_{-1}[D^{i2}_{tS_1}F] - k_1[D_{tS_1}][F] + k_{-1}[D^{e2}_{tS_1}F] - k_1[D_{tS_1}][F] + k_{-1}[d^{i2}_{tS_1}f] - k_1[D_{tS_1}][f] + k_{-1}[d^{e2}_{tS_1}f] - k_1[D_{tS_1}][f], \\
\frac{d[D^{i2}_{tS_1}F]}{dt} &= k_1[D_{tS_1}][F] - k_{-1}[D^{i2}_{tS_1}F] + k_{-2}[D^{i2}_{tS_1}F_{2,0}] - k_2[D^{i2}_{tS_1}F][F] + k_{-1}[D^{i2}_{tS_1}F_{1,1}] - k_1[D^{i2}_{tS_1}F][F], \\
\frac{d[D^{i2}_{tS_1}F_{2,0}]}{dt} &= k_2[D^{i2}_{tS_1}F][F] - k_{-2}[D^{i2}_{tS_1}F_{2,0}] + k_{-1}[D^{i2}_{tS_1}F_3] - k_1[D^{i2}_{tS_1}F_{2,0}][F], \\
\frac{d[D^{i2}_{tS_1}F_{1,1}]}{dt} &= k_1[D^{i2}_{tS_1}F][F] - k_{-1}[D^{i2}_{tS_1}F_{1,1}] + k_{-2}[D^{i2}_{tS_1}F_3] - k_2[D^{i2}_{tS_1}F_{1,1}][F], \\
\frac{d[D^{i2}_{tS_1}F_3]}{dt} &= k_1[D^{i2}_{tS_1}F_{2,0}][F] - k_{-1}[D^{i2}_{tS_1}F_3] + k_2[D^{i2}_{tS_1}F_{1,1}][F] - k_{-2}[D^{i2}_{tS_1}F_3] + k_{-2}[D^{i2}_{tS_1}F_4] - k_2[D^{i2}_{tS_1}F_3][F], \\
\frac{d[D^{i2}_{tS_1}F_4]}{dt} &= k_2[D^{i2}_{tS_1}F_3][F] - k_{-2}[D^{i2}_{tS_1}F_4] + k_{-3}[H^{i2}_5] - k_3[D^{i2}_{tS_1}F_4], \\
\frac{d[D^{e2}_{tS_1}F]}{dt} &= k_1[D_{tS_1}][F] - k_{-1}[D^{e2}_{tS_1}F] + k_{-2}[D^{e2}_{tS_1}F_{2,0}] - k_2[D^{e2}_{tS_1}F][F] + k_{-1}[D^{e2}_{tS_1}F_{1,1}] - k_1[D^{e2}_{tS_1}F][F], \\
\frac{d[D^{e2}_{tS_1}F_{2,0}]}{dt} &= k_2[D^{e2}_{tS_1}F][F] - k_{-2}[D^{e2}_{tS_1}F_{2,0}] + k_{-1}[D^{e2}_{tS_1}F_3] - k_1[D^{e2}_{tS_1}F_{2,0}][F], \\
\frac{d[D^{e2}_{tS_1}F_{1,1}]}{dt} &= k_1[D^{e2}_{tS_1}F][F] - k_{-1}[D^{e2}_{tS_1}F_{1,1}] + k_{-2}[D^{e2}_{tS_1}F_{1,1}] - k_2[D^{e2}_{tS_1}F][F], \\
\frac{d[D^{e2}_{tS_1}F_3]}{dt} &= k_1[D^{e2}_{tS_1}F_{2,0}][F] - k_{-1}[D^{e2}_{tS_1}F_3] + k_2[D^{e2}_{tS_1}F_{1,1}][F] - k_{-2}[D^{e2}_{tS_1}F_3] + k_{-2}[D^{e2}_{tS_1}F_4] - k_2[D^{e2}_{tS_1}F_3][F], \\
\frac{d[D^{e2}_{tS_1}F_4]}{dt} &= k_2[D^{e2}_{tS_1}F_3][F] - k_{-2}[D^{e2}_{tS_1}F_4] + k_{-3}[H^{e2}_1] - k_3[D^{e2}_{tS_1}F_4], \\
\frac{d[H^{i1}_1]}{dt} &= k_3[D^{i1}_{S_1}F_4] - k_{-3}[H^{i1}_1] - k_4[H^{i1}_1] + k_{-4}[H^{i1}_2], \\
\frac{d[H^{i1}_2]}{dt} &= k_4[H^{i1}_1] - k_{-4}[H^{i1}_2] - k_5[H^{i1}_2] + k_{-5}[H^{i1}_3], \\
\frac{d[H^{i1}_3]}{dt} &= k_5[H^{i1}_2] - k_{-5}[H^{i1}_3] - k_6[H^{i1}_3] + k_{-6}[H^{i1}_4], \\
\frac{d[H^{i1}_4]}{dt} &= k_6[H^{i1}_3] - k_{-6}[H^{i1}_4] - k_7[H^{i1}_4] + k_{-7}[H^{i1}_5],, \\
\frac{d[H^{i1}_5]}{dt} &= k_7[H^{i1}_4] - k_{-7}[H^{i1}_5] - k_{-3}[H^{i1}_5] + k_3[D^{i1}_{tS_2}F_4], \\
\frac{d[H^{i2}_1]}{dt} &= k_3[D^{i2}_{S_1}F_4] - k_{-3}[H^{i2}_1] - k_4[H^{i2}_1] + k_{-4}[H^{i2}_2], \\
\frac{d[H^{i2}_2]}{dt} &= k_4[H^{i2}_1] - k_{-4}[H^{i2}_2] - k_5[H^{i2}_2] + k_{-5}[H^{i2}_3], \\
\frac{d[H^{i2}_3]}{dt} &= k_5[H^{i2}_2] - k_{-5}[H^{i2}_3] - k_6[H^{i2}_3] + k_{-6}[H^{i2}_4], \\
\frac{d[H^{i2}_4]}{dt} &= k_6[H^{i2}_3] - k_{-6}[H^{i2}_4] - k_7[H^{i2}_4] + k_{-7}[H^{i2}_5], \\
\frac{d[H^{i2}_5]}{dt} &= k_7[H^{i2}_4] - k_{-7}[H^{i2}_5] - k_{-3}[H^{i2}_5] + k_3[D^{i2}_{tS_1}F_4], \\
\frac{d[H^{e1}_1]}{dt} &= k_3[D^{e1}_{tS_2}F_4] - k_{-3}[H^{e1}_1] - k_4[H^{e1}_1] + k_{-4}[H^{e1}_2], \\
\frac{d[H^{e1}_2]}{dt} &= k_4[H^{e1}_1] - k_{-4}[H^{e1}_2] - k_5[H^{e1}_2] + k_{-5}[H^{e1}_3], \\
\frac{d[H^{e1}_3]}{dt} &= k_5[H^{e1}_2] - k_{-5}[H^{e1}_3] - k_6[H^{e1}_3] + k_{-6}[H^{e1}_4], \\
\frac{d[H^{e1}_4]}{dt} &= k_6[H^{e1}_3] - k_{-6}[H^{e1}_4] - k_7[H^{e1}_4] + k_{-7}[H^{e1}_5], \\
\frac{d[H^{e1}_5]}{dt} &= k_7[H^{e1}_4] - k_{-7}[H^{e1}_5] - k_{-3}[H^{e1}_5] + k_3[D^{e1}_{S_2}F_2][D^{e1}_{S_X}F_2], \\
\frac{d[H^{e2}_1]}{dt} &= k_3[D^{e2}_{tS_1}F_4] - k_{-3}[H^{e2}_1] - k_4[H^{e2}_1] + k_{-4}[H^{e2}_2], \\
\frac{d[H^{e2}_2]}{dt} &= k_4[H^{e2}_1] - k_{-4}[H^{e2}_2] - k_5[H^{e2}_2] + k_{-5}[H^{e2}_3],, \\
\frac{d[H^{e2}_3]}{dt} &= k_5[H^{e2}_2] - k_{-5}[H^{e2}_3] - k_6[H^{e2}_3] + k_{-6}[H^{e2}_4], \\
\frac{d[H^{e2}_4]}{dt} &= k_6[H^{e2}_3] - k_{-6}[H^{e2}_4] - k_7[H^{e2}_4] + k_{-7}[H^{e2}_5], \\
\frac{d[H^{e2}_5]}{dt} &= k_7[H^{e2}_4] - k_{-7}[H^{e2}_5] - k_{-3}[H^{e2}_5] + k_3[D^{e2}_{S_2}F_2][D^{e2}_{S_X}F_2],
\end{aligned}
$$

$$
\begin{aligned}
\frac{d[D_{S_1}]}{dt} &= k_{-1}[D^{i1}_{S_1}F] - k_1[D_{S_1}][F] + k_{-1}[D^{i2}_{S_1}F] - k_1[D_{S_1}][F] + k_{-1}[d^{i1}_{S_1}f] - k_1[D_{S_1}][f] + k_{-1}[d^{i2}_{S_1}f] - k_1[D_{S_1}][f],\\
\frac{d[D^{i1}_{S_1}F]}{dt} &= k_1[D_{S_1}][F] - k_{-1}[D^{i1}_{S_1}F] + k_{-2}[D^{i1}_{S_1}F_{2,0}] - k_2[D^{i1}_{S_1}F][F] + k_{-1}[D^{i1}_{S_1}F_{1,1}] - k_1[D^{i1}_{S_1}F][F],\\
\frac{d[D^{i1}_{S_1}F_{2,0}]}{dt} &= k_2[D^{i1}_{S_1}F][F] - k_{-2}[D^{i1}_{S_1}F_{2,0}] + k_{-1}[D^{i1}_{S_1}F_3] - k_1[D^{i1}_{S_1}F_{2,0}][F],\\
\frac{d[D^{i1}_{S_1}F_{1,1}]}{dt} &= k_1[D^{i1}_{S_1}F][F] - k_{-1}[D^{i1}_{S_1}F_{1,1}] + k_{-2}[D^{i1}_{S_1}F_3] - k_2[D^{i1}_{S_1}F_{1,1}][F],\\
\frac{d[D^{i1}_{S_1}F_3]}{dt} &= k_1[D^{i1}_{S_1}F_{2,0}][F] - k_{-1}[D^{i1}_{S_1}F_3] + k_2[D^{i1}_{S_1}F_{1,1}][F] - k_{-2}[D^{i1}_{S_1}F_3] + k_{-2}[D^{i1}_{S_1}F_4] - k_2[D^{i1}_{S_1}F_3][F],\\
\frac{d[D^{i1}_{S_1}F_4]}{dt} &= k_2[D^{i1}_{S_1}F_3][F] - k_{-2}[D^{i1}_{S_1}F_4] + k_{-3}[H^{i1}_1] - k_3[D^{i1}_{S_1}F_4],\\
\frac{d[D^{i2}_{S_1}F]}{dt} &= k_1[D_{S_1}][F] - k_{-1}[D^{i2}_{S_1}F] + k_{-2}[D^{i2}_{S_1}F_{2,0}] - k_2[D^{i2}_{S_1}F][F] + k_{-1}[D^{i2}_{S_1}F_{1,1}] - k_1[D^{i2}_{S_1}F][F],\\
\frac{d[D^{i2}_{S_1}F_{2,0}]}{dt} &= k_2[D^{i2}_{S_1}F][F] - k_{-2}[D^{i2}_{S_1}F_{2,0}] + k_{-1}[D^{i2}_{S_1}F_3] - k_1[D^{i2}_{S_1}F_{2,0}][F],\\
\frac{d[D^{i2}_{S_1}F_{1,1}]}{dt} &= k_1[D^{i2}_{S_1}F][F] - k_{-1}[D^{i2}_{S_1}F_{1,1}] + k_{-2}[D^{i2}_{S_1}F_3] - k_2[D^{i2}_{S_1}F_{1,1}][F],\\
\frac{d[D^{i2}_{S_1}F_3]}{dt} &= k_1[D^{i2}_{S_1}F_{2,0}][F] - k_{-1}[D^{i2}_{S_1}F_3] + k_2[D^{i2}_{S_1}F_{1,1}][F] - k_{-2}[D^{i2}_{S_1}F_3] + k_{-2}[D^{i2}_{S_1}F_4] - k_2[D^{i2}_{S_1}F_3][F],\\
\frac{d[D^{i2}_{S_1}F_4]}{dt} &= k_2[D^{i2}_{S_1}F_3][F] - k_{-2}[D^{i2}_{S_1}F_4] + k_{-3}[H^{i2}_1] - k_3[D^{i2}_{S_1}F_4],\\
\frac{d[D_{S_2}]}{dt} &= k_{-1}[D^{e1}_{S_2}F] - k_1[D_{S_2}][F] + k_{-1}[D^{e2}_{S_2}F] - k_1[D_{S_2}][F] + k_{-1}[d^{e1}_{S_2}F] - k_1[D_{S_2}][F] + k_{-1}[d^{e2}_{S_2}F] - k_1[D_{S_2}][F],\\
\frac{d[D^{e1}_{S_2}F]}{dt} &= k_1[D_{S_2}][F] - k_{-1}[D^{e1}_{S_2}F] + k_{-2}[D^{e1}_{S_2}F_2] - k_2[D^{e1}_{S_2}F][F],\\
\frac{d[D^{e1}_{S_2}F_2]}{dt} &= k_2[D^{e1}_{S_2}F][F] - k_{-2}[D^{e1}_{S_2}F_2] + k_{-3}[H^{e1}_5] - k_3[D^{e1}_{S_2}F_2],\\
\frac{d[D^{e2}_{S_2}F]}{dt} &= k_1[D_{S_2}][F] - k_{-1}[D^{e2}_{S_2}F] + k_{-2}[D^{e2}_{S_2}F_2] - k_2[D^{e2}_{S_2}F][F],\\
\frac{d[D^{e2}_{S_2}F_2]}{dt} &= k_2[D^{e2}_{S_2}F][F] - k_{-2}[D^{e2}_{S_2}F_2] + k_{-3}[H^{e2}_5] - k_3[D^{e2}_{S_2}F_2],\\
\frac{d[D_{S_X}]}{dt} &= k_{-1}[D^{e1}_{S_X}F] - k_1[D_{S_X}][F] + k_{-1}[D^{e2}_{S_X}F] - k_1[D_{S_X}][F] + k_{-1}[d^{e1}_{S_X}F] - k_1[D_{S_X}][F] + k_{-1}[d^{e2}_{S_X}F] - k_1[D_{S_X}][F] - \delta[D_{S_X}],\\
\frac{d[D^{e1}_{S_X}F]}{dt} &= k_1[D_{S_X}][F] - k_{-1}[D^{e1}_{S_X}F] + k_{-2}[D^{e1}_{S_X}F_2] - k_2[D^{e1}_{S_X}F][F],\\
\frac{d[D^{e1}_{S_X}F_2]}{dt} &= k_2[D^{e1}_{S_X}F][F] - k_{-2}[D^{e1}_{S_X}F_2] + k_{-3}[H^{e1}_5] - k_3[D^{e1}_{S_X}F_2],\\
\frac{d[D^{e2}_{S_X}F]}{dt} &= k_1[D_{S_X}][F] - k_{-1}[D^{e2}_{S_X}F] + k_{-2}[D^{e2}_{S_X}F_2] - k_2[D^{e2}_{S_X}F][F],\\
\frac{d[D^{e2}_{S_X}F_2]}{dt} &= k_2[D^{e2}_{S_X}F][F] - k_{-2}[D^{e2}_{S_X}F_2] + k_{-3}[H^{e2}_5] - k_3[D^{e2}_{S_X}F_2],\\
\frac{d[D_{tS_2}]}{dt} &= k_{-1}[D^{i1}_{tS_2}F] - k_1[D_{tS_2}][F] + k_{-1}[D^{e1}_{tS_2}F] - k_1[D_{tS_2}][F] + k_{-1}[d^{i1}_{tS_2}F] - k_1[D_{tS_2}][F] + k_{-1}[d^{e1}_{tS_2}F] - k_1[D_{tS_2}][F],\\
\frac{d[D^{i1}_{tS_2}F]}{dt} &= k_1[D_{tS_2}][F] - k_{-1}[D^{i1}_{tS_2}F] + k_{-2}[D^{i1}_{tS_2}F_{2,0}] - k_2[D^{i1}_{tS_2}F][F] + k_{-1}[D^{i1}_{tS_2}F_{1,1}] - k_1[D^{i1}_{tS_2}F][F],\\
\frac{d[D^{i1}_{tS_2}F_{2,0}]}{dt} &= k_2[D^{i1}_{tS_2}F][F] - k_{-2}[D^{i1}_{tS_2}F_{2,0}] + k_{-1}[D^{i1}_{tS_2}F_3] - k_1[D^{i1}_{tS_2}F_{2,0}][F],\\
\frac{d[D^{i1}_{tS_2}F_{1,1}]}{dt} &= k_1[D^{i1}_{tS_2}F][F] - k_{-1}[D^{i1}_{tS_2}F_{1,1}] + k_{-2}[D^{i1}_{tS_2}F_3] - k_2[D^{i1}_{tS_2}F_{1,1}][F],\\
\frac{d[D^{i1}_{tS_2}F_3]}{dt} &= k_1[D^{i1}_{tS_2}F_{2,0}][F] - k_{-1}[D^{i1}_{tS_2}F_3] + k_2[D^{i1}_{tS_2}F_{1,1}][F] - k_{-2}[D^{i1}_{tS_2}F_3] + k_{-2}[D^{i1}_{tS_2}F_4] - k_2[D^{i1}_{tS_2}F_3][F],\\
\frac{d[D^{i1}_{tS_2}F_4]}{dt} &= k_2[D^{i1}_{tS_2}F_3][F] - k_{-2}[D^{i1}_{tS_2}F_4] + k_{-3}[H^{i1}_5] - k_3[D^{i1}_{tS_2}F_4],\\
\frac{d[D^{e1}_{tS_2}F]}{dt} &= k_1[D_{tS_2}][F] - k_{-1}[D^{e1}_{tS_2}F] + k_{-2}[D^{e1}_{tS_2}F_{2,0}] - k_2[D^{e1}_{tS_2}F][F] + k_{-1}[D^{e1}_{tS_2}F_{1,1}] - k_1[D^{e1}_{tS_2}F][F],\\
\frac{d[D^{e1}_{tS_2}F_{2,0}]}{dt} &= k_2[D^{e1}_{tS_2}F][F] - k_{-2}[D^{e1}_{tS_2}F_{2,0}] + k_{-1}[D^{e1}_{tS_2}F_3] - k_1[D^{e1}_{tS_2}F_{2,0}][F],\\
\frac{d[D^{e1}_{tS_2}F_{1,1}]}{dt} &= k_1[D^{e1}_{tS_2}F][F] - k_{-1}[D^{e1}_{tS_2}F_{1,1}] + k_{-2}[D^{e1}_{tS_2}F_3] - k_2[D^{e1}_{tS_2}F_{1,1}][F],\\
\frac{d[D^{e1}_{tS_2}F_3]}{dt} &= k_1[D^{e1}_{tS_2}F_{2,0}][F] - k_{-1}[D^{e1}_{tS_2}F_3] + k_2[D^{e1}_{tS_2}F_{1,1}][F] - k_{-2}[D^{e1}_{tS_2}F_3] + k_{-2}[D^{e1}_{tS_2}F_4] - k_2[D^{e1}_{tS_2}F_3][F],\\
\frac{d[D^{e1}_{tS_2}F_4]}{dt} &= k_2[D^{e1}_{tS_2}F_3][F] - k_{-2}[D^{e1}_{tS_2}F_4] + k_{-3}[H^{e1}_1] - k_3[D^{e1}_{tS_2}F_4].
\end{aligned}
$$

Table 2: Mechanistic model ODEs derived from biochemical equations through the application of mass action kinetics.

Table S3: Optimal parameter set (mechanistic model)

Parameter	Value ($M^{-1}s^{-1}$)	Parameter	Value (s^{-1})	Parameter	Value (Ms^{-1})
k_1 (i_1,e_2)	0.1322	k_{-1} (i_1,e_2)	0.9584	α_a	0.5950
k_2 (i_1,e_2)	0.4585	k_{-2} (i_1,e_2)	0.8997	α_b	0.7735
k_1 (i_2,e_1)	0.2639	k_{-1} (i_2,e_1)	0.9835	–	–
k_2 (i_2,e_1)	0.9152	k_{-2} (i_2,e_1)	0.9232	–	–
k_3	0.2495	k_{-3}	0.9563	–	–
k_4	0.3160	k_{-4}	0.0652	–	–
k_5	0.8690	k_{-5}	0.8571	–	–
k_6	0.3282	k_{-6}	0.3254	–	–
k_7	0.8436	k_{-7}	0.8657	–	–
k_8	0.4897	k_{-8}	0.1614	–	–
K_a	0.7693	K_b	0.4187	–	–
–	–	β_p	0.7793	–	–
–	–	β_d	0.3725	–	–
–	–	δ	0.9733	–	–

Table 3: The optimal mechanistic model parameter values inferred by the genetic algorithm through global optimisation. Model parameters are dimensional, taking SI units arising from standard mass action kinetics.

Table S4: Optimal parameter set (reduced model)

Parameter	Value ($M^{-1}s^{-1}$)	Parameter	Value (s^{-1})	Parameter	Value (Ms^{-1})
k_1	0.9956	K_b	0.0108	α_a	0.0063
k_2	0.8365	β_p	0.6494	α_b	0.0001
k_3	0.9984	β_d	0.9935	–	–
k_4	0.9950	δ	0.0215	–	–
k_r	0.3525	–	–	–	–
K_a	0.0036	–	–	–	–

Table 4: The optimal reduced model parameter values inferred by the genetic algorithm through global optimisation. Model parameters are dimensional, taking SI units arising from standard mass action kinetics.

Section S1: Conservation relations

To demonstrate how the factor of a half arises in our ODE for State 2, consider the simple biochemical reaction equation:

$$A \underset{k_{-1}}{\overset{k_1}{\rightleftharpoons}} B + C. \tag{1}$$

That is, a reaction in which one molecule (A) forms two distinct products (B, C) and both products must combine in order to reproduce the original molecule. The application of mass action kinetics produces the following ODE model:

$$\frac{dA}{dt} = k_{-1}BC - k_1A, \tag{2}$$
$$\frac{dB}{dt} = k_1A - k_{-1}BC, \tag{3}$$
$$\frac{dC}{dt} = k_1A - k_{-1}BC. \tag{4}$$

From this we can derive the following conservation relations:

$$\frac{dA}{dt} + \frac{dB}{dt} = 0, \tag{5}$$
$$\frac{dA}{dt} + \frac{dC}{dt} = 0. \tag{6}$$

From (5) we have

$$\int \left(\frac{dA}{dt} + \frac{dB}{dt}\right) dt = \int 0 \; dt, \tag{7}$$
$$\implies \quad A + B = c, \quad \text{(constant of integration)} \tag{8}$$
$$\implies \quad c = A_0, \quad (\text{at } t = 0,\ A = A_0,\ B = 0) \tag{9}$$
$$\implies \quad A + B = A_0. \tag{10}$$

Similarly, from (6)

$$A + C = A_0. \tag{11}$$

Hence, adding (10) and (11) gives

$$2A + B + C = 2A_0, \tag{12}$$
$$\implies \quad A + \frac{B}{2} + \frac{C}{2} = A_0. \tag{13}$$

Therefore, in order to preserve the initial concentration of the molecule A (A_0), we must impose the factor of a half in our ODE for State 2. The corresponding numerical solutions will therefore satisfy the desired relationship that the summed concentrations of all model variables at any time point t will equal the initial concentration of model variables present when $t = 0$. This preserves the conservation of mass for the excision reactions which give rise to two products: the final genetic state (State 2) and the circle of DNA.
